# Improving Estimates of Social Contact Patterns for Airborne Transmission of Respiratory Pathogens

**DOI:** 10.3201/eid2810.212567

**Published:** 2022-10

**Authors:** Nicky McCreesh, Mbali Mohlamonyane, Anita Edwards, Stephen Olivier, Keabetswe Dikgale, Njabulo Dayi, Dickman Gareta, Robin Wood, Alison D. Grant, Richard G. White, Keren Middelkoop

**Affiliations:** London School of Hygiene and Tropical Medicine TB Centre, London, UK (N. McCreesh, A.D. Grant, R.G. White);; The Desmond Tutu HIV Centre, University of Cape Town, Cape Town, South Africa (M. Mohlamonyane, R. Wood, K. Middelkoop);; University of KwaZulu-Natal Africa Health Research Institute, Durban, South Africa (A. Edwards, A.D. Grant);; Africa Health Research Institute, Durban (S. Olivier, K. Dikgale, N. Dayi, D. Gareta);; University of Cape Town Department of Medicine, Cape Town (R. Wood, K. Middelkoop);; University of the Witwatersrand School of Public Health, Johannesburg, South Africa (A.D. Grant)

**Keywords:** respiratory infections, tuberculosis and other mycobacteria, social contact, age-mixing, airborne, mathematical modelling, tuberculosis, South Africa

## Abstract

Data on social contact patterns are widely used to parameterize age-mixing matrices in mathematical models of infectious diseases. Most studies focus on close contacts only (i.e., persons spoken with face-to-face). This focus may be appropriate for studies of droplet and short-range aerosol transmission but neglects casual or shared air contacts, who may be at risk from airborne transmission. Using data from 2 provinces in South Africa, we estimated age mixing patterns relevant for droplet transmission, nonsaturating airborne transmission, and *Mycobacterium tuberculosis* transmission, an airborne infection where saturation of household contacts occurs. Estimated contact patterns by age did not vary greatly between the infection types, indicating that widespread use of close contact data may not be resulting in major inaccuracies. However, contact in persons >50 years of age was lower when we considered casual contacts, and therefore the contribution of older age groups to airborne transmission may be overestimated.

Mathematical models of infectious disease transmission are widely used to help develop infectious disease policy, estimate the potential effect of interventions, and provide insight into disease dynamics and natural history. Many models incorporate patterns of mixing between different sections of the population, most commonly between different age groups. Simulated mixing patterns can have a considerable effect on model dynamics (*1*), underscoring the importance of simulating realistic mixing patterns. Mixing patterns are frequently shaped by social contact data (i.e., empirical data collected from respondents about the persons with whom they had contact during a set period) (*2*).

Most social contact data collection has focused on close contacts, using a definition of contacts that required a 2-way face-to-face conversation of >3 words, close proximity (e.g., within 2 meters), physical contact, or some combination of those criteria (*2*). Those types of contact may approximate reasonably well the types of contact that are relevant for infections that are transmitted primarily through direct contact, short range aerosols, droplets, or some combination of these modes. For obligate, preferential, or opportunistic airborne infections such as measles, *Mycobacterium tuberculosis*, and SARS-CoV-2, however, this definition probably excludes many potentially effective contacts because transmission of airborne infections can occur between anybody sharing air in inadequately ventilated indoor spaces, regardless of whether conversation occurs, and over distances >2 meters (*3*). For airborne infections, estimates of casual contact time may therefore be more appropriate, calculated as the time spent in indoor locations multiplied by the number of other persons present.

Tuberculosis also differs from most respiratory infections in terms of the long periods during which persons are potentially infectious; an estimated 9–36 months elapses between disease development and diagnosis (or notification) in 11 countries with high tuberculosis incidences (*4*). Therefore, transmission to repeated contacts can partially saturate (even allowing for reinfection), making the relationship between contact time and infection risk nonlinear (*5*). This effect is most pronounced for contact between household members (*5*). Household membership and repeated contacts are rarely explicitly simulated in mathematical models, and therefore the effects of contact saturation need to be incorporated into the mixing matrices used to parameterize the models.

In this article, we describe methods for estimating age-mixing patterns relevant for nonsaturating airborne transmission and *M. tuberculosis* transmission by using a novel weighted approach to incorporate the effects of household contact saturation into our estimates for *M. tuberculosis*. We generate estimates of age mixing using data on close and casual contacts from 2 communities in South Africa and compare the estimated mixing patterns with those typically used in mathematical modeling studies (i.e., generated using close contact numbers, and more suitable for droplet or short range aerosol transmission).

## Methods

We collected social contact data in 2 study communities in South Africa: 1 in KwaZulu-Natal Province and 1 in Western Cape Province. Both communities have high rates of unemployment, high prevalence of HIV, and high incidence of tuberculosis compared with the other provinces as a whole. The study community in KwaZulu-Natal consisted of a population of ≈46,000, living in the predominantly rural and peri-urban areas in the catchment areas of 2 primary care clinics and within a demographic surveillance area (DSA). The study community in Western Cape was a peri-urban community of ≈27,000 and was an established research site with biennial censuses.

### Data Collection

We collected the KwaZulu-Natal data during March–December 2019. We sampled 3,093 adults (>18 years of age) at random from an estimated population of 33,288, stratified by residential area (small-scale divisions with ≈350 households per area) and with probability proportional to the number of eligible persons in each area, based on the most recent DSA census conducted before area entry. We made up to 3 attempts to contact sampled persons.

We collected the Western Cape data during May–October 2019. In total, we selected 1,530 adults (>15 years of age) from an estimated population of 20,633, by using age- and sex-stratified random sampling, based on a census conducted in the study population in February and March 2019. We made up to 5 attempts to contact selected persons on different days of the week (including weekends).

For both surveys, we conducted interviews face-to-face at the respondents’ homes, by using interview administered questionnaires on tablet computers. We conducted interviews in isiZulu in KwaZulu-Natal and in English or isiXhosa in Western Cape. We asked respondents about their movements on a randomly assigned day during the preceding week in KwaZulu-Natal, and on the day before the interview in Western Cape. To allow casual contact time (defined as time spent “sharing air” indoors or on transport) to be estimated, we asked respondents to list the places they had visited (including their own home) and transport they had used. For each location, questions asked included:

- What type of location was it? (Appendix Figure 5)
- How long did you spend there? (recorded in hours and minutes)
- How many persons were there halfway through the time you were there?

We did not ask respondents for the ages of persons present because it was thought that respondents would not be able to accurately remember and estimate the ages of all persons present in all indoor locations visited and transport used. We also asked respondents about their close contacts, defined as persons with whom the respondent had a face-to-face conversation. We first asked respondents to make a numbered list of all their contacts, with help from the interviewer. We then asked respondents questions about 10 contacts (selected at random by number by the tablet computers) or all of their contacts if they reported <10. Questions included:

- Is this contact a member of your household?
- How old do you think they are?
- How much time did you spend with them in total?

We also collected respondents’ basic demographic information. For the KwaZulu-Natal community, we obtained data on household size and residency (i.e., urban, peri-urban, or rural) from the most recent DSA census. We collected all other data directly from the respondents.

### Data Analysis

We estimated close contact numbers and times by using data on persons with whom the respondents reported having a face-to-face conversation. We generated 95% plausible intervals for the age-mixing matrices by using bootstrapping.

We estimated casual contact time in a location as the duration of time the respondent reported spending there multiplied by the reported number of persons present. We generated central estimates for casual contact time age-mixing matrices by using the method outlined in McCreesh et al (*6*). In brief, because data were collected on numbers of total persons and children present in indoor locations only, and not the ages of adults, we need to estimate the age distribution of adult casual contacts. We therefore assumed that the age distribution of adult contacts in each location type matched the weighted age distribution of respondents who reported visiting locations of that type. Again, we generated 95% plausible ranges by using bootstrapping.

We adjusted the age-mixing matrices to be symmetric by using the study community age structures. We used data on adult contact numbers and time with children to estimate child contact numbers and time with adults, assuming that overall contact numbers and time between children and adults in each age group is equal to overall contact numbers and time between adults in each age group and children. To enable comparison between the 2 study communities, the lowest respondent age group was set at 15–19 years for both surveys. Because persons 15–17 years of age were not interviewed in KwaZulu-Natal, we assumed that contact patterns in persons 18–19 years of age were representative of contact patterns in all persons 15–19 years of age (Appendix).

### Generating Age-Mixing Matrices for Droplet and Nonsaturating Airborne Transmission and *Mycobacteria tuberculosis*

**Figure 1:**
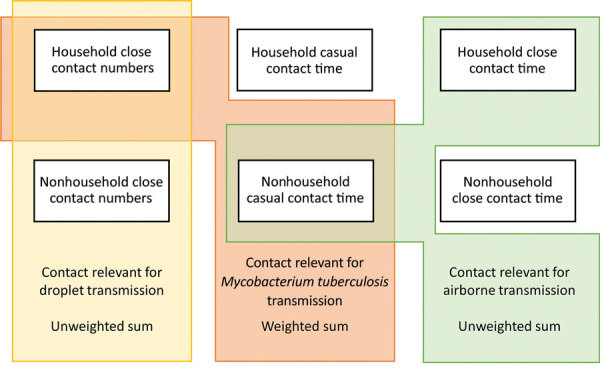
Summary of data used to estimate age-mixing matrices for a study of social contact patterns for airborne transmission of respiratory pathogens, KwaZulu Natal and Western Cape Provinces, South Africa, 2019. Diagram showing how age-mixing matrices relevant for the transmission of droplet infections, airborne infections, and *Mycobacterium tuberculosis* were estimated using empirical data on close contact numbers, close contact time, and casual contact time.

We set age-mixing matrices relevant for droplet transmission to be equal to age-mixing matrices calculated using close contact numbers (Figure 1). We set age-mixing matrices relevant for nonsaturating airborne transmission to be equal to the unweighted sum of the household close contact time matrices and the nonhousehold casual contact time matrices. We used close contact time between household members for household estimates, as opposed to casual contact time occurring in households. We did so because most contact between household members is likely to meet the definition of close contact, and because this approach enabled the age structures of households to be more accurately reflected in the age-mixing matrices. We set age-mixing matrices relevant for *M. tuberculosis* transmission to be equal to the sum of the household close contact number matrices and the nonhousehold casual contact time matrices. We weighted these matrices to reflect empirical estimates of the proportion of tuberculosis that results from household transmission (central estimate 12% [range 8%–16%]) (*5*).

To enable direct comparisons to be made between the different age-mixing matrices, we adjusted the matrices for nonsaturating airborne transmission and *M. tuberculosis* transmission to give the same mean contact intensity between adults as the matrices for droplet transmission. We used bootstrapping to generate plausible ranges (Appendix). 

## Results

### Recruitment

Of the 3,093 persons sampled in KwaZulu-Natal, 1,723 (56%) were successfully contacted, 299 (10%) were dead or reported to have out-migrated, and 1,071 (35%) could not be contacted. Of those successfully contacted, 1,704 (99%) completed an interview.

Of the 1,530 persons sampled in Western Cape, 1,214 (93%) were successfully contacted, 117 (8%) had moved or died, 193 (13%) had had incorrect information listed in the census, and 6 were uncontactable. Of the 1,214 persons contacted, 77 (6%) refused to be interviewed and 14 were ineligible (because of disability or lack of fluency with English and isiXhosa). Of 1,123 persons interviewed, unexplained technical issues meant that data from 8 interviews were lost between collection and transfer to the database, leaving 1,115 (92%) completed interviews.

For both populations, the recruited sample was a reasonable match to the target population in terms of sex, age, and residence type (urban, peri-urban, or rural) (Table). Respondents in Kwa-Zulu-Natal also were a close match to the target population in terms of employment status (Appendix). No data on employment status for the target population were available for Western Cape.

**Table:** Characteristics of respondents and target population for study of social contact patterns for airborne transmission of respiratory pathogens, KwaZulu Natal and Western Cape Provinces, South Africa, 2019

Characteristic	KwaZulu Natal			Western Cape	
	Sample, no. (%)	Target population, %*		Sample, no. (%)	Target population, %*
Sex					
M	751 (44)	41		553 (50)	52
F	953 (56)	59		562 (50)	48
Age group, y					
15–17	0	9.1		56 (5)	4.5
18–19	118 (6.9)	5.6		84 (7.5)	4.5
20–29	495 (29)	26		412 (37)	33
30–39	308 (18)	21		358 (32)	37
40–49	227 (13)	13		142 (13)	15
>50	556 (33)	25		63 (5.7)	6.5
Residence					
Rural	867 (51)	59		0	0
Peri-urban	716 (42)	33		1,115 (100)	100
Urban	121 (7.1)	8		0	0
Monthly household income, South African rands					
<1,000	416 (24)			111 (10)	
1,000–2,500	785 (46)			261 (23)	
2,500–5,000	302 (18)			374 (34)	
5,000–10,000	125 (7.3)			179 (16)	
>10,000	65 (3.8)			61 (5.5)	
Unknown/missing	11 (0.65)			129 (12)	
Employment					
Full-time	329 (19)			403 (36)	
Part-time/casual	68 (4)			213 (19)	
None	1299 (76)			492 (44)	
Missing	8 (0.5)			7 (0.6)	
Household size					
1	115 (6.7)	4.1		203 (18)	19
2–4	287 (17)	26		683 (61)	66
5–7	488 (29)	33		195 (17)	13
8–10	375 (22)	20		26 (2.3)	1.6
>11	439 (26)	17		8 (0.72)	0.4
Day reported					
Monday	239 (14)			203 (18)	
Tuesday	242 (14)			202 (18)	
Wednesday	239 (14)			187 (17)	
Thursday	251 (15)			138 (12)	
Friday	261 (15)			80 (7.2)	
Saturday	245 (14)			98 (8.8)	
Sunday	227 (13)			207 (19)	
Total	1,704	33,288		1,115	20,633

*Target population refers to persons in the populations >15 years of age. †In KwaZulu-Natal, urban is defined as KwaMsane Municipality, peri-urban as other areas with a population density >400/km^2^, and rural as areas with a population density <400/km^2^.

### Contact Numbers and Time

**Figure 2:**
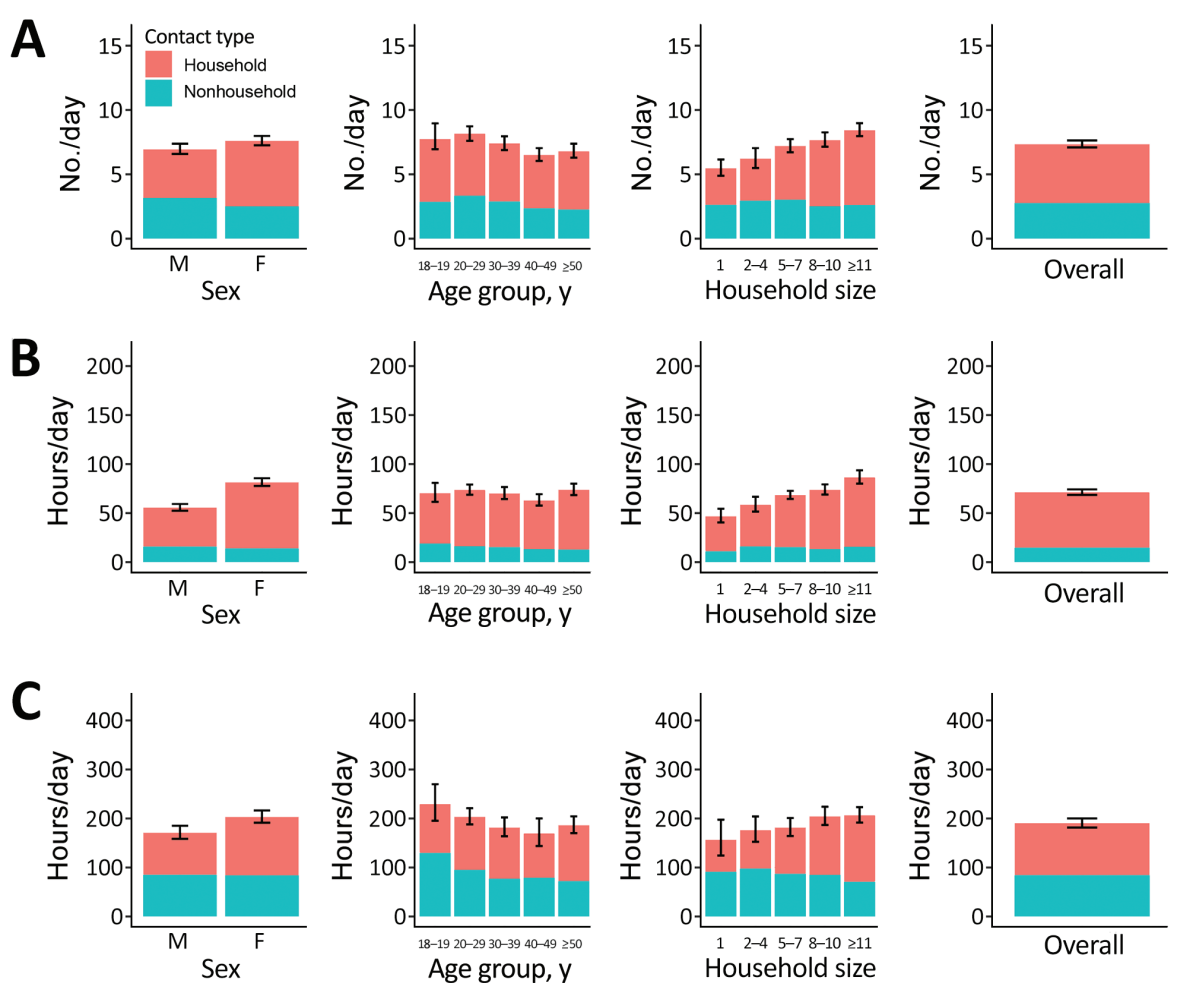
Household and nonhousehold close contact numbers (A), close contact time (B), and casual contact time (C) for study of social contact patterns for airborne transmission of respiratory pathogens, KwaZulu-Natal Province, South Africa, by sex, age group, and household size. Error bars show 95% CIs for total contact numbers or time. For KwaZulu-Natal, household size data were taken from census data and did not always correspond exactly with respondents’ views of who they considered to be household members. For this reason, some contact with household members was reported by respondents who we recorded as having a household size of 1.

**Figure 3:**
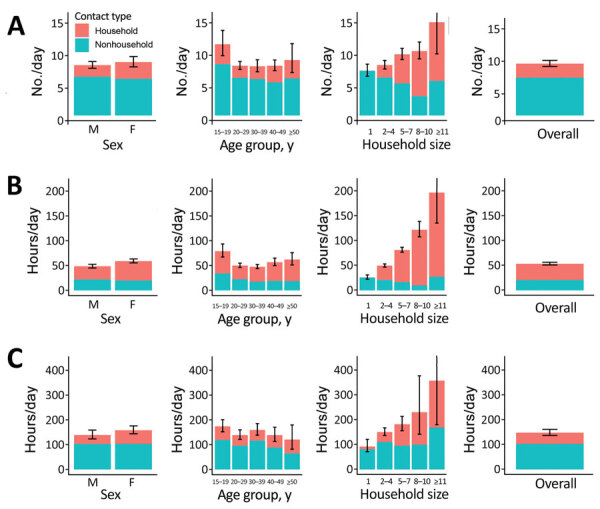
Household and nonhousehold close contact numbers (A), close contact time (B), and casual contact time (C) in Western Cape Province, South Africa, by sex, age, and household size, for study of social contact patterns for airborne transmission of respiratory pathogens. Error bars show 95% CIs for total contact numbers or time. In Western Cape, contact with household members was reported by a small proportion of respondents who had reported having no household members, most likely reflecting errors in the data.

We stratified household and nonhousehold close contact numbers and time and casual contact time in KwaZulu-Natal and Western Cape, by sex, age, and household size (Figure 2, 3; Appendix Tables 1–6). Overall, close contact numbers and time, as well as casual contact time, were higher for women than for men in both communities; however, the differences were generally not large (close contact time 46% higher for women in KwaZulu-Natal and 8%–22% higher for other contact measures and settings) and not significant for close contact numbers or casual contact time in Western Cape. We observed a tendency for casual contact time to decrease slightly with age in both communities, and close contact numbers and time were substantially higher in persons 15–19 years of age than in older age groups in Western Cape only (Western Cape close contact numbers: 11 in persons 15–19 years of age, 7.7–8.7 in older age groups [p<0.001]; close contact time: 80 hours in persons 15–19 years of age, 49–63 in older age groups [p<0.001]). Close contact numbers and time, as well as casual contact time, increased with increasing household size in both communities, driven by increases in contact with household members. Contact between household members made up a higher proportion of total contact in KwaZulu-Natal than in Western Cape for all types of contact (close contact numbers: 62% in KwaZulu-Natal, 27% in Western Cape; close contact time: 79% in KwaZulu-Natal, 60% in Western Cape; casual contact time: 55% in KwaZulu-Natal, 31% in Western Cape).

### Age Mixing

**Figure 4:**
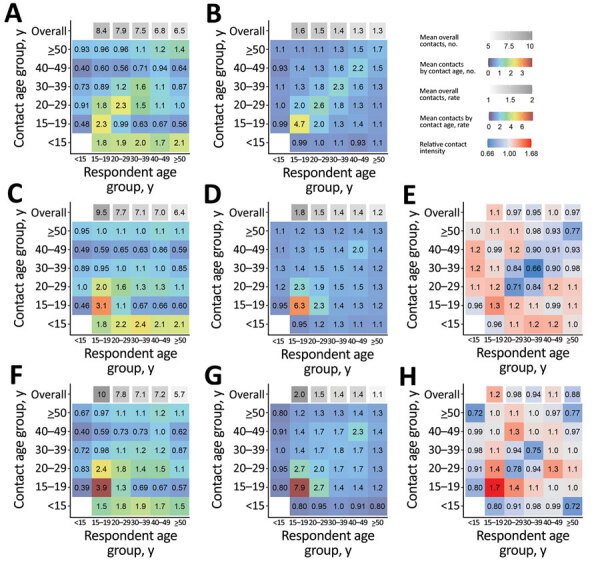
Age-mixing matrices relevant for droplet transmission (A,B), nonsaturating airborne transmission (C,D), and *Mycobacterium tuberculosis* transmission (F,G) for study of social contact patterns for airborne transmission of respiratory pathogens, KwaZulu-Natal Province, South Africa. Panels A, C, and F show absolute contact intensities between respondents and contacts in each age group; panels B, D, and G show intensities of contact between each member of each age group; panels E and H show intensities for airborne infections and *M. tuberculosis* compared with intensities for droplet infections, respectively. Numbers shown in panel A are the mean number of contacts respondents in each age group have with contacts in each age group per day. Numbers shown in panel B are the rate of contact between each person in the population per day, expressed as rates × 10^5^. Numbers and rates in panels C, D, F, and G are standardized so that the mean overall contact intensity by reported by adult respondents is the same as the mean number of overall close contacts reported by adult respondents (panel A). Contact numbers between child respondents and contacts in each age group were estimated from data on contact between adult respondents and child contacts.

**Figure 5:**
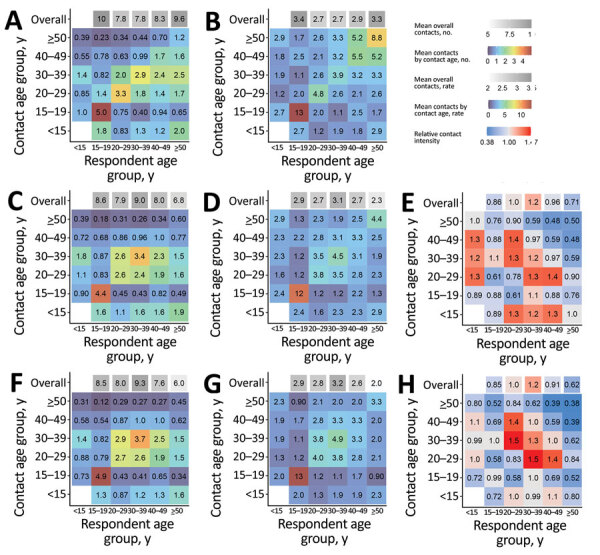
Age-mixing matrices relevant for droplet transmission (A,B), nonsaturating airborne transmission (C,D), and *Mycobacterium tuberculosis* transmission (F,G) for study of social contact patterns for airborne transmission of respiratory pathogens, Western Cape Province, South Africa. Panels A, C, and F show absolute contact intensities between respondents and contacts in each age group; panels B, D, and G show intensities of contact between each member of each age group; panels E and H show intensities for airborne infections and *Mycobacterium tuberculosis* compared with intensities for droplet infections, respectively. Numbers shown in panel A are the mean number of contacts respondents in each age group have with contacts in each age group per day. Numbers shown in panel B are the rate of contact between each person in the population per day, expressed as rates × 10^5^. Numbers and rates in panels C, D, F, and G are standardized so that the mean overall contact intensity by reported by adult respondents is the same as the mean number of overall close contacts reported by adult respondents (panel A). Contact numbers between child respondents and contacts in each age group were estimated from data on contact between adult respondents and child contacts.

We generated estimated age-mixing matrices for droplet transmission non-saturating airborne transmission, and *M. tuberculosis* transmission for KwaZulu-Natal and Western Cape (Figure 4, 5). We also generated 95% plausible ranges for these matrices (Appendix Figure 1, 2).

Estimated contact patterns by age did not vary greatly between the infection types in either community. However, age-mixing patterns were less assortative in the nonsaturating airborne and *M. tuberculosis* matrices compared with the droplet matrices in both communities (Appendix). The exception to this pattern was contact between persons 15–19 years of age in KwaZulu-Natal, which was more intense in the nonsaturating airborne and *M. tuberculosis* matrices than the droplet matrices. In both communities, relative to other adult age groups, overall contact intensities were lower in persons >50 years of age when considering contact relevant for nonsaturating airborne transmission or the transmission of *M. tuberculosis* compared with contact relevant for droplet transmission.

## Discussion

Using data from 2 provinces in South Africa, we estimated contact and age-mixing patterns relevant for the transmission of droplet infections, nonsaturating airborne infections, and *M. tuberculosis.* In our communities, contact patterns did not vary greatly between contacts relevant for droplet infections and those relevant for nonsaturating airborne or *M. tuberculosis* transmission. However, using close contact data in models of the transmission of *M. tuberculosis* or other airborne infections in our study communities may mean that the importance of adults >50 years of age to transmission is overestimated.

Very few data are available on casual contact patterns from any setting. Previous studies in the same community in Western Cape have found greater drops in casual contact time than in close contact numbers in older age groups (*6*) and decreases in indoor casual contact numbers with age (*7*). Another study in the same community found high levels of age-assortative mixing with respect to casual contact time in schools and workplaces (*8*). More data are needed on casual contact patterns, and age-mixing patterns in particular, to determine whether the findings of this study are generalizable to other settings and to improve the predictions from mathematical models of the transmission of *M. tuberculosis* and other airborne infections.

Our approaches to generating the separate droplet and airborne transmission matrices are necessarily simplifications, and many infections will not fit neatly into these 2 categories. In addition, considerable uncertainty exists about the role of different transmission routes to the spread of many infections. Droplets have traditionally been considered to be the main transmission route for most respiratory viruses; however, there is evidence that airborne transmission can occur for a wide range of pathogens, including influenza, respiratory syncytial virus, Middle East respiratory syndrome coronavirus, and SARS-CoV-2 (*9*). One model using data on household transmission of influenza A suggested that airborne transmission was responsible for about half of infections (*10*). For infections where both airborne and droplet or short range aerosol transmission are thought to play an important role in transmission, an intermediate matrix may be preferable.

There are 2 main differences between our droplet and airborne or *M. tuberculosis* age-mixing matrices. The first is the type of nonhousehold contacts considered: close (face-to-face conversation) or casual (sharing space indoors). The second is that the airborne and (nonhousehold component of the) *M. tuberculosis* matrices are based on contact time, rather than unique contact numbers. The primary reason for using contact time for casual contacts is that respondents are unlikely to be able to estimate unique casual contact numbers for many locations they visit, necessitating the use of contact time or assumptions about the rate of turnover of unique persons in a location. For our droplet transmission matrices, we chose to use unique contact numbers in a 24-hour period because that is the most commonly used method (*2*) and therefore enables comparisons to be made with what is typically done. However, we should note that both the choice of a 24-hour time period and the lack of any weighting or restrictions by contact duration or other measures of closeness are relatively arbitrary choices.

Robust evidence as to the types of contact most relevant to transmission are limited for respiratory infections. Several studies have compared the fit to data on varicella, parvovirus B19, or influenza A seroprevalence by age of models parameterized by using contact patterns generated from close contact data in a range of different ways (*11*–*13*). Overall, those studies suggest that analysis methods that give greater weight to more intimate contacts may be preferable in some circumstances; for instance, restricting what counts as a contact to those involving physical touch or a minimum contact duration or using contact time rather than contact numbers. Approaches based on contact numbers may be more suitable for more highly transmissible infections such as measles, where only a short duration of contact is needed for transmission, whereas approaches based on contact time may be more suitable for less transmissible infections, where repeated or longer contacts are needed (*14*).

Fewer studies have considered expanding the pool of contacts beyond close contacts only, to also include casual contacts. However, a study that had paired individual-level contact data and pandemic influenza A serologic data found that models that included a variable for number of locations visited were strongly supported over those that only included variables for age and close contact numbers (*15*). This finding suggests that airborne transmission may play a role in the spread of influenza A, or that the standard close contact definition misses a substantial proportion of contacts at risk for droplet transmission.

Other factors may also influence airborne and *M. tuberculosis* transmission risk, which are not accounted for in the analyses. Ventilation rates play a large role in determining airborne infection risk (*16*), and giving less weight to contact occurring in better ventilated settings would improve our airborne and *M. tuberculosis* matrices. Unfortunately, few data on ventilation rates by location type are available, and they show large amounts of variation between locations and between the same location on different days (*17*). Saturation of contacts may occur for infections other than *M. tuberculosis*, particularly highly transmissible pathogens such as measles virus. An approach based on casual contact numbers may be preferable for these infections but would be highly dependent on assumptions made about how unique contact numbers are related to estimates of cross-sectional numbers of persons present.

There are several limitations when using casual contact data to estimate mixing patterns. First, estimates of contact time in places where large numbers of persons are present are likely to be less reliable because a person’s estimates of the number of persons present are likely to be poor and because the assumption that a risk for transmission exists between all persons present in the space may not be true in larger spaces. Estimates may be poorer when asking about a random day in the past week (as we did in KwaZulu-Natal) than when asking about the day before the interview (as we did in Western Cape). In our main analysis, when estimating contact time, we cap the number of persons at risk for transmission at 100. In our sensitivity analyses, we show that using a cap of 20 persons or not capping the numbers of persons has a moderate effect on casual contact time age-mixing matrices (Appendix). Conducting similar sensitivity analyses may be necessary when using age-mixing matrices calculated using casual contact time in mathematical models.

A second limitation is that the approach we use to determining the ages of adults present in locations other than respondents’ own homes is indirect and relies on the assumption that the age distribution of adults present in a location type reflects the duration of time respondents of different ages reported spending in that location type. This assumption may not always be reasonable if different age groups tend to visit different locations of the same type (or at different times) or substantial mixing occurs with persons from outside the study community. These issues are discussed further in McCreesh et al. (*6*)*.*

An additional limitation of our estimates for KwaZulu-Natal only is that we did not recruit persons 15–17 years of age and instead assumed in the analysis that contact by persons 18–19 years of age was representative of contact by all persons 15–19 years of age. This assumption is unlikely to be true given that contacts by persons 15–17 and 18–19 years of age differ greatly in Western Cape (Appendix Figure 9). For this reason, our estimates for persons 15–19 years of age in KwaZulu-Natal should be treated with caution. 

To conclude, our estimated age-mixing matrices for droplet transmission, nonsaturating airborne transmission, and *M. tuberculosis* transmission were not substantially different from each other for either community. This finding provides some reassurance that the widespread use of close contact data to parameterize age-mixing matrices for transmission models of airborne infections may not be resulting in major inaccuracies. Some differences were observed, however, particularly in the oldest age group, and our data were from 2 communities in South Africa only. We recommend that future social contact surveys collect data on casual contacts as well as close contacts to determine whether the similarity between different types of contact pattern is true across other settings. We would also urge mathematical modelers to consider whether unique close contact numbers in a 24-hour period are the most appropriate contacts for the infection and scenario they are simulating and to consider performing sensitivity analyses when uncertainty exists as to the most appropriate contact definition.

Appendix Additional information about improving estimates of social contact patterns for airborne transmission of respiratory pathogens.
