# Supplementary material for: Improving Estimates of Social Contact Patterns for Airborne Transmission of Respiratory Pathogens
Source: Emerg Infect Dis. 2022 Oct;28(10):2016–26. doi: 10.3201/eid2810.212567 (PMC9514345; doi:10.3201/eid2810.212567)
Supplement: Appendix — Additional information about improving estimates of social contact patterns for airborne transmission of respiratory pathogens. [file 21-2567-Techapp-s1.pdf]

Article DOI: https://doi.org/10.3201/eid2810.212567

Improving Estimates of Social Contact Patterns for Airborne Transmission of Respiratory Pathogens

Appendix

Methods

Ethics

Ethical approval for the data collection in KwaZulu-Natal was granted by the Biomedical Research Ethics Committee (BREC) of the University of KwaZulu-Natal (BE662/17) and the London School of Hygiene & Tropical Medicine Observational / Interventions Research Ethics Committee (14640). Ethical approval for the data collection in Western Cape was granted by the Human Research Ethics Committee at the University of Cape Town (HREC/REF: 008/2018) and the London School of Hygiene & Tropical Medicine Observational / Interventions Research Ethics Committee (14520). Informed consent was obtained from all participants.

Comparison of employment status with target population, KwaZulu-Natal

To investigate the whether we may have under-recruited people who were employed in KwaZulu-Natal, we compared data on employment from the most recent DSA survey between respondents in the social contact survey and the target population aged 18+ years as a whole. No data on employment status for the target population were available for Western Cape.

Weighting

All analyses of contact numbers and contact time were weighted. For KwaZulu-Natal, they were weighted to the study population composition by age group (18–19, 20–29, 30–39, 40–49, 50+) and sex. For Western Cape, they were weighted to the study population composition by age group (15–17, 18–19, 20–29, 30–39, 40–49, 50+) and sex. As fewer respondents were asked about Fridays and Saturdays, the Western Cape data were also weighted by the day of the week.

Estimating close contact numbers

Respondents were first asked to make a numbered list of all their contacts, with help from the interviewer. The total number of contacts was recorded on the tablet computers, along with the number of those contacts who were members of the respondent's household. Close contact numbers by respondent characteristic were estimated using the total number of close contacts that the respondent reported, and the number of those contacts who were household members. Respondents who reported more household contacts than total contacts were excluded from the analysis.

Close contact age mixing matrices were generated using data on close contacts on which more detailed information were available (all contacts if respondents reported ≤10, a random 10 if they reported more). When generating the central estimate, contact numbers by age group for respondents were multiplied by T / D_a, where T was the total number of contacts that they reported, and D_a was the total number of contacts whose age was provided. Respondents who reported a nonzero number of close contacts, but who did not give the age of any of their contacts were excluded. 95% plausible intervals were generated using 10,000 bootstrapped samples, re-sampling respondents with replacement within age categories, and re-sampling *T* contacts with replacement from the set of all contacts of the respondent on which detailed information were collected (*1*). Only sampled contacts who were or weren't reported to be a member of the respondent's household were included when estimating household and 'other' age-mixing patterns respectively.

The age mixing matrices were adjusted to be symmetric, using the study community age structures. Data on adult contacts with children were used to estimate child contact with adults. To allow comparison between the two study communities, and between close and casual age mixing patterns, the lowest respondent age group was set at 15–19 years for both surveys. As 15–17 year olds were not interviewed in KwaZulu-Natal, we assumed that contact patterns in 18–19 years olds were representative of contact patterns in all 15–19 year olds, and adjusted the weights accordingly.

Estimating close contact time

The approach used for estimating close contact time was the same as that used for estimating close contact numbers, except that contact numbers were multiplied by the amount of time that respondents reported spending with each contact that day.

Contacts with the contact duration missing were excluded when generating bootstrap samples for estimating close contact time age mixing patterns. When generating the central estimate, contact numbers by age group for respondents were multiplied by T / D_{ad}, where T was the total number of contacts that they reported, and D_{ad} was the total number of contacts whose age and duration were both provided.

Estimating casual contact time

For each location visited, respondents were asked to identify the location type, from a list of frequently visited location types identified by local researchers and fieldworkers before the start of data collection. If the interviewer could not identify the correct location type on the list, they selected 'Other' and gave details. In the analysis, locations were excluded if it was considered likely that most all or of the time would have been spent outdoors (e.g., if the details given were 'gardening'). Several responses were re-coded, if it was considered plausible from the details given that the location was covered by one of the original options (e.g., 'domestic worker' was changed to 'House off plot'). Several new location categories were added, if reported by multiple respondents (e.g., 'factory'). Finally, remaining responses in the 'Other' category were recorded as 'Other' if the type of location could be determined from the free text variable, and 'Missing' if it could not be.

Respondents were excluded from all casual contact time analyses if:

1) They reported visiting no locations (including their own home) and using no transport
2) The variable giving the total number locations visited was missing, and no information was provided on any locations visited
3) No information was available on any of the locations visited or transport used

Casual contact time was estimated as the duration of time that respondents reported spending in a location, multiplied by the number of people that they estimated were present at

the location, halfway through the time that they were there. In the analysis, total numbers of people present were capped at a maximum of 100, as above this value, it is unlikely that the respondent had sufficient contact with each person present to allow transmission. Estimates of numbers of adults and children present were reduced by the same proportion for each location, to give a maximum total number of people present of 100.

If the estimated number of people or children present was missing for a location, or if the estimated number of children present was greater than the estimated total number of people present, then the numbers of adults and children present were set equal to the mean reported number of adults and children present at locations of that type in the same community (KwaZulu-Natal or Western Cape). These numbers were rounded to the nearest whole number when generating bootstrap samples. If the duration of time spent in the location was missing, the duration was set equal to the mean duration for locations of that type.

Age mixing matrices for casual contact time were generated using the data on locations visited, the duration of time spent in the location, and the estimated number of adults and children present. Central estimates were generated using the method outlined in McCreesh *et al* (*2*). As data were collected on numbers of people and children present in indoor locations only, and not on the ages of adults present, the age distribution of adult casual contacts needed to be estimated. To do so, we assumed that the age distribution of adults present in each location type matched the age distribution of respondents who reported visiting locations of that type, weighted by the duration of time they reported spending in that location type and weighted to the sampled population age and sex distribution. To generate plausible ranges, 10,000 bootstrap samples were generated, re-sampling respondents with replacement within age categories, and sampling the ages of adults present by resampling with replacement respondents who reported visiting locations of that type (weighted by duration, and to the sampled age and sex distribution). The number of children present was set equal to number of children present reported by the respondent. Contact times were then estimated by multiplying the duration of time the respondent reported spending in each location by the sampled number of people of each age group present.

The age mixing matrices were made symmetric, using data on adult contact time with children to estimate child contact time with adults. As respondents were asked to estimate

numbers of children present who were aged under 15 years, we set the lowest respondent age group to be 15–19 years. As 15–17 year olds were not interviewed in KwaZulu-Natal, we assumed that contact patterns in 18–19 years olds were representative of contact patterns in all 15–19 year olds, and adjusted the weights accordingly.

Estimating age-mixing patterns relevant for the transmission of droplet infections

Age-mixing patterns relevant for the transmission of droplet infections were assumed to be equal to age mixing patterns calculated from close contact numbers.

Estimating age-mixing patterns relevant for the transmission of airborne infections

To generate age-mixing patterns relevant for the transmission of airborne infections, we summed estimated close contact time between household members, and estimated casual contact time occurring in locations other than the respondents' own houses. 95% plausible ranges were generated by pairing each of the 10,000 close contact household bootstrapped matrices with one of the 10,000 outside-household casual contact time bootstrapped matrices.

To allow direct comparisons to be made between the different age mixing matrices, the matrices for airborne infections (central estimate and 10,000 individual bootstrapped matrices) were then adjusted to give the same mean contact intensity between adults as the matrices for close contacts (using the central estimate matrices and 10,000 individual bootstrapped matrices respectively).

Estimating age-mixing patterns relevant for the transmission of *Mycobacterium tuberculosis*

It is estimated that, in high incidence settings, only 8%–19% of tuberculosis comes from household transmission (*2*). Long durations of disease (*3*) also mean that transmission to household members may partially saturate, meaning that the relationship between contact time and transmission is nonlinear for household contacts. We therefore estimated age mixing matrices relevant to the transmission of *Mtb* by creating weighted averages of close contact numbers with household members, and casual contact time occurring outside respondents' own households.

For each pair of bootstrapped household close contact number and non-household casual contact time matrices, a proportion of contact that should occur in households was sampled from a uniform distribution between 8%–16% (the range of values found by different studies), with

12% used for the central estimate. A weighted average was then generated, given the desired proportion of overall 'contact' by adults occurring in households.

To allow direct comparisons to be made between the different age mixing matrices, the matrices for *Mtb* (central estimate and 10,000 individual bootstrapped matrices) were then adjusted to give the same mean contact intensity between adults as the matrices for close contacts (using the central estimate matrices and 10,000 individual bootstrapped matrices respectively).

Assortative mixing by age group

We quantified the assortativeness of mixing by age group using the index Q, which takes the value of 1 when all contact occurs within age groups, and 0 when there is homogeneous mixing by age (*4*). 95% plausible intervals and p-values for the difference between assortativeness in the airborne and *Mtb* transmission matrices compared to the droplet transmission matrices were generated using the bootstrapped samples.

Sensitivity analyses

In our main analysis, we cap the number of people at risk of infection in locations (i.e., casual contact numbers) at a maximum of 100. In the sensitivity analysis, we explore the effect of setting the cap at 20, or not having a cap.

When generating the non-saturating airborne and *Mtb* age-mixing matrices, we used close contact time and close contact number data respectively to estimate contact time between household members. In the sensitivity analysis, we explore the effect of using casual contact time.

For all sensitivity analysis age-mixing matrices, we rescaled contact times to give the same overall mean contact hours per adult in the sensitivity analysis as in the main analysis. This was done because the primary use of age mixing matrices is in mathematical modeling, where it is usually the relative values of the cells in the matrices that has an impact on model dynamics, not the absolute values.

Results

Comparison of employment status with target population, KwaZulu-Natal

19.3% (329/1704) of social contact survey respondents gave their employment status as ‘Full time’ in the DSA census, compared to 19.5% (5898/30259) of the target population; and 4.0% (69/1704) of respondents gave their employment status as ‘Part time’, compared to 3.8% (1139/30259) of the target population.

Missing/incomplete data

Close contact data

- The reported number of household contacts was higher than the reported total number for two respondents in KwaZulu-Natal. They were excluded from all analyses of close contacts.
- Three respondents in KwaZulu-Natal had the contact age missing for all of their contacts, and were excluded from the age-mixing analysis.
- The number of household contacts was unknown for 11 respondents in KwaZulu-Natal. They were not included in the analysis of household or non-household contacts (Appendix Figures 10–13).
- Contact ages were missing for 54 contacts in KwaZulu-Natal and 16 contacts in Western Cape
- Whether a contact is a household member was missing for 16 contacts in KwaZulu-Natal and two in Western Cape
- Contact durations were missing for 16 contacts in KwaZulu-Natal and three contacts in Western Cape. No respondents were missing the duration of time for all contacts.

Casual contact data

- One person in KwaZulu-Natal and eight in Western Cape reported that they visited no locations (including their own house) and used no transport used. This was considered to be implausible, and they were excluded from the casual contact time analyses.

- The ‘total number of locations visited’ variable was missing for four respondents in Western Cape, and they gave no information on any locations visited. They were excluded from the casual contact time analyses.
- 43 respondents in Western Cape reported the total number of locations visited, but did not give any information on any of the locations visited. They did not differ from other respondents in Western Cape in respect to the reported number of locations visited (no details given: mean locations = 3.0, 95% CI 2.5–3.5, detail given: 3.0 (2.9–3.1), and were excluded from the casual contact time analyses.
- The number of people and/or number of children present was missing for 102 locations in KwaZulu-Natal and 220 in Western Cape. The reported number of children present was greater than reported total number of people present for seven locations in KwaZulu-Natal and none in Western Cape.
- The duration of time spent in a location was missing for two locations in Western Cape and none in KwaZulu-Natal.

Assortative mixing by age group

Using the data from KwaZulu-Natal, the index of assortativeness Q took the value of 0.14 (95% plausible range 0.11 – 0.17) for the droplet transmission matrix, 0.10 (0.08–0.12) for the airborne transmission matrix ($p = 0.01$, when compared to the droplet transmission matrix), and 0.12 (0.09 – 0.13) for the Mtb transmission matrix ($p = 0.09$). Using the data from Western Cape, the index of assortativeness Q took the value of 0.24 (95% plausible range 0.20 – 0.28) for the droplet transmission matrix, 0.21 (0.17–0.25) for the airborne transmission matrix ($p = 0.13$), and 0.23 (0.19 – 0.26) for the Mtb transmission matrix ($p = 0.24$).

Sensitivity analyses

Changing the cap on people at risk in locations

Overall casual contact time was lower when the number of people at risk was capped at 20, and higher when it was not capped, compared to when it was capped at 100 people (Appendix Figure 1). Changing the cap had a moderate effect on casual contact time age-mixing patterns, although most changes were not large compared to the breadths of the 95% plausible ranges (Appendix Figure 2).

Generating age-mixing matrices using casual contact time for household contact

Calculating age-mixing matrices relevant for non-saturating airborne and *Mtb* transmission using casual contact time data for contact in between household members had very little effect on estimated age-mixing patterns (Supporting information, Appendix Figures 5, 6). The exception to this was contact relevant to airborne transmission between 15–19 year olds in KwaZulu-Natal, which was lower when casual contact time data were used than in the main analysis.

References

1. Funk S. socialmixr: social mixing matrices for infectious disease modelling. Version 0.1.8. 2020 [cited 2021 Jan 1]. https://cran.r-project.org/web/packages/socialmixr/index.html

2. McCreesh N, Morrow C, Middelkoop K, Wood R, White RG. Estimating age-mixing patterns relevant for the transmission of airborne infections. Epidemics. 2019;28:100339. PubMed https://doi.org/10.1016/j.epidem.2019.03.005

3. Ku C-C, MacPherson P, Khundi M, Nzawa R, Feasey HR, Nliwasa M, et al. Estimated durations of asymptomatic, symptomatic, and care-seeking phases of tuberculosis disease. BMC Med. 2021;19:298. PubMed https://doi.org/10.1186/s12916-021-02128-9

4. Gupta S, Anderson RM, May RM. Networks of sexual contacts: implications for the pattern of spread of HIV. AIDS. 1989;3:807–17. PubMed https://doi.org/10.1097/00002030-198912000-00005

Appendix Table 1. Mean overall, household, and non-household close contact numbers in KwaZulu-Natal. P-values were calculated using a Wald test.

		Overall		Household members		Non-household members	
Characteristic		Mean (95% CI)	p-value	Mean (95% CI)	p-value	Mean (95% CI)	p-value
Sex	Male	7.0 (6.6–7.4)	0.02	3.8 (3.6–4.0)	<0.001	3.2 (2.8–3.5)	0.007
	Female	7.6 (7.3–8.0)		5.1 (4.9–5.3)		2.5 (2.2–2.9)	
Age	15–19	7.9 (7.0–9)	<0.001	4.9 (4.3–5.5)	0.08	2.9 (2.1–3.8)	0.01
	20–29	8.2 (7.6–8.7)		4.8 (4.5–5.1)		3.3 (2.9–3.9)	
	30–39	7.4 (6.9–8.0)		4.5 (4.2–4.9)		2.9 (2.5–3.4)	
	40–49	6.5 (6.0–7.0)		4.2 (3.8–4.6)		2.4 (2.0–2.8)	
	50+	6.8 (6.3–7.4)		4.5 (4.2–4.8)		2.3 (1.8–2.8)	
Residence	Peri-Urban	7.0 (6.6–7.5)	0.1	4.3 (4.0–4.5)	<0.001	2.7 (2.4–3.1)	0.1
	Urban	7.6 (7.3–8.0)		5.0 (4.8–5.2)		2.6 (2.4–2.9)	
	Rural	7.4 (6.0–9.1)		3.5 (3.1–3.9)		3.9 (2.7–5.8)	
Monthly household income	<R1000	6.8 (6.3–7.4)	0.003	4.2 (4.0–4.5)	<0.001	2.5 (2.1–3.1)	0.09
	R1000–R2500	7.6 (7.2–7.9)		5.0 (4.8–5.3)		2.6 (2.3–2.9)	
	R2500–R5000	7.2 (6.5–7.9)		4.2 (3.8–4.5)		3.1 (2.5–3.8)	
	R5000–R10000	7.1 (6.3–8.0)		4.2 (3.7–4.9)		2.9 (2.3–3.6)	
	>R10000	9.7 (7.5–13)		4.6 (3.8–5.7)		5.1 (3–8.6)	
	Unknown/missing	5.5 (4.3–7.0)		2.5 (1.8–3.4)		3.0 (2.0–4.3)	
Employment	Full-time	7.4 (6.7–8.2)	0.9	3.8 (3.5–4.2)	<0.001	3.6 (2.9–4.4)	0.007
	Part-time	7.1 (6.1–8.2)		4.0 (3.3–4.8)		3.0 (2.4–3.8)	
	None/missing	7.4 (7.1–7.6)		4.8 (4.6–5.0)		2.5 (2.3–2.8)	
Household size	1	5.5 (4.9–6.2)	<0.001	2.9 (2.5–3.3)	<0.001	2.6 (2.1–3.3)	0.5
	2–4	6.2 (5.5–7.0)		3.3 (2.9–3.6)		3.0 (2.3–3.8)	
	5–7	7.2 (6.7–7.7)		4.2 (4.0–4.4)		3.0 (2.6–3.6)	
	8–10	7.7 (7.1–8.3)		5.2 (4.9–5.4)		2.5 (2.1–3.0)	
	11+	8.5 (8.0–9.0)		5.8 (5.4–6.2)		2.6 (2.3–3.0)	
Day reported	Sunday	7.6 (6.8–8.4)	0.2	4.5 (4.1–4.9)	0.9	3.2 (2.5–4.0)	0.005
	Monday	7.5 (6.7–8.4)		4.6 (4.2–5.1)		2.9 (2.2–3.8)	
	Tuesday	6.9 (6.4–7.4)		4.8 (4.4–5.2)		2.0 (1.7–2.4)	
	Wednesday	7.2 (6.7–7.7)		4.6 (4.2–5.0)		2.6 (2.2–3.0)	
	Thursday	8.1 (7.3–9.1)		4.6 (4.2–5.1)		3.4 (2.7–4.4)	
	Friday	7.0 (6.5–7.7)		4.5 (4.1–4.9)		2.6 (2.1–3.1)	
	Saturday	7.2 (6.5–7.9)		4.5 (4.1–4.9)		2.7 (2.2–3.4)	
Total		7.4 (7.1–7.6)		4.6 (4.4–4.7)		2.8 (2.5–3.0)	

Appendix Table 2. Mean overall, household, and non-household close contact numbers in Western Cape. P-values were calculated using a Wald test.

		Overall		Household members		Non-household members	
Characteristic		Mean (95% CI)	p-value	Mean (95% CI)	p-value	Mean (95% CI)	p-value
Sex	Male	7.9 (7.3–8.4)	0.3	1.8 (1.7–2.0)	<0.001	6.0 (5.5–6.6)	0.5
	Female	8.3 (7.6–9.2)		2.6 (2.5–2.8)		5.7 (5.0–6.6)	
Age	15–19	11 (9.3–13)	0.004	3.1 (2.8–3.5)	<0.001	8.0 (6.3–10)	0.05
	20–29	7.8 (7.2–8.4)		1.9 (1.7–2.1)		5.9 (5.3–6.5)	
	30–39	7.7 (6.8–8.7)		2.0 (1.8–2.2)		5.7 (4.8–6.7)	
	40–49	7.8 (7.0–8.6)		2.6 (2.3–3.0)		5.2 (4.4–6.0)	
	50+	8.7 (6.7–11)		2.9 (2.4–3.5)		5.8 (3.8–8.7)	
Residence	Peri-Urban	8.1 (7.6–8.6)		2.2 (2.1–2.3)		5.9 (5.4–6.4)	
	Urban	NA		NA		NA	
	Rural	NA		NA		NA	
Monthly household income	<R1000	9.2 (6.5–13)	0.07	2.0 (1.7–2.5)	<0.001	7.2 (4.6–11)	0.03
	R1000–R2500	7.8 (7.1–8.6)		2.1 (1.8–2.3)		5.7 (5.1–6.5)	
	R2500–R5000	8.6 (8.0–9.3)		2.2 (2–2.4)		6.4 (5.8–7.1)	
	R5000–R10000	7.2 (6.3–8.2)		2.0 (1.8–2.3)		5.2 (4.3–6.2)	
	>R10000	8.5 (6.7–11)		2.7 (2.3–3.2)		5.8 (4.1–8.2)	
	Unknown/missing	7.1 (6.2–8.1)		2.7 (2.4–3.1)		4.4 (3.5–5.4)	
Employment	Full-time	8.3 (7.7–9.0)	0.3	2.0 (1.9–2.2)	<0.001	6.3 (5.7–7.0)	0.03
	Part-time	8.5 (7.1–10)		1.9 (1.7–2.2)		6.6 (5.2–8.4)	
	None/missing	7.6 (7.0–8.3)		2.5 (2.4–2.7)		5.1 (4.5–5.8)	

Characteristic		Overall Mean (95% CI)	Overall p-value	Household members Mean (95% CI)	Household members p-value	Non-household members Mean (95% CI)	Non-household members p-value
Household size	1	7.0 (6.1–8.0)	<0.001	0.05 (0.02–0.12)	<0.001	7.0 (6.1–7.9)	<0.001
	2–4	7.9 (7.2–8.6)		1.9 (1.9–2.0)		5.9 (5.3–6.6)	
	5–7	9.5 (8.7–11)		4.5 (4.4–4.7)		5.0 (4.2–6.0)	
	8–10	10 (8.8–11)		7.0 (6.3–7.9)		3.0 (2.0–4.5)	
	11+	15 (9.6–22)		9.1 (6.7–13)		5.4 (1.9–15)	
Day reported	Sunday	8.1 (7.3–8.9)	0.5	2.0 (1.8–2.3)	0.08	6.1 (5.3–6.9)	0.8
	Monday	8.6 (7.7–9.6)		2.4 (2.2–2.7)		6.2 (5.3–7.3)	
	Tuesday	7.8 (7.0–8.8)		2.2 (1.9–2.5)		5.6 (4.8–6.6)	
	Wednesday	8.5 (7.3–9.9)		2.2 (2.0–2.5)		6.2 (5.1–7.7)	
	Thursday	8.0 (7.0–9.1)		2.0 (1.8–2.4)		5.9 (5.0–7.0)	
	Friday	7.1 (6.1–8.3)		2.0 (1.6–2.4)		5.2 (4.2–6.3)	
	Saturday	8.5 (6.5–11)		2.6 (2.2–3.0)		5.9 (4.1–8.7)	
Total		8.1 (7.6–8.6)		2.2 (2.1–2.3)		5.9 (5.4–6.4)	

Appendix Table 3. Mean overall, household, and non-household close contact time (hours) in KwaZulu-Natal. P-values were calculated using a Wald test.

Characteristic		Overall Mean (95% CI)	Overall p-value	Household members Mean (95% CI)	Household members p-value	Non-household members Mean (95% CI)	Non-household members p-value
Sex	Male	56 (52–59)	<0.001	40 (37–43)	<0.001	16 (14–18)	0.2
	Female	82 (78–86)		67 (64–71)		14 (12–16)	
Age	15–19	71 (62–81)	0.08	52 (42–63)	0.07	19 (15–25)	0.1
	20–29	74 (69–79)		58 (53–63)		16 (14–18)	
	30–39	70 (64–77)		55 (49–61)		15 (13–18)	
	40–49	63 (58–69)		50 (44–56)		13 (11–16)	
	50+	74 (68–80)		61 (56–66)		13 (10–17)	
Residence	Peri-Urban	70 (66–75)	0.01	54 (50–59)	<0.001	16 (13–18)	0.4
	Urban	74 (70–78)		60 (56–64)		14 (13–15)	
	Rural	60 (52–68)		43 (37–51)		16 (12–22)	
Monthly household income	<R1000	72 (66–78)	<0.001	61 (56–67)	<0.001	11 (9–13)	<0.001
	R1000–R2500	81 (77–86)		69 (65–74)		12 (10–13)	
	R2500–R5000	53 (48–59)		33 (30–37)		20 (16–26)	
	R5000–R10000	56 (50–63)		30 (25–37)		26 (22–31)	
	>R10000	73 (62–85)		41 (32–53)		32 (24–41)	
	Unknown/missing	34 (28–40)		23 (16–34)		10 (6–18)	
Employment	Full-time	64 (58–71)	0.05	45 (40–50)	<0.001	19 (15–25)	0.02
	Part-time	67 (54–82)		51 (38–67)		16 (12–21)	
	None/missing	73 (70–77)		60 (57–63)		14 (12–15)	
Household size	1	47 (40–54)	<0.001	36 (30–43)	<0.001	11 (8–15)	0.2
	2–4	59 (52–67)		43 (37–49)		16 (11–22)	
	5–7	68 (65–73)		53 (49–57)		15 (13–18)	
	8–10	74 (69–79)		61 (56–66)		13 (11–16)	
	11+	87 (80–94)		71 (64–78)		16 (14–18)	
Day reported	Sunday	71 (64–78)	0.8	56 (49–63)	0.9	15 (12–18)	0.4
	Monday	70 (62–79)		53 (47–61)		17 (12–25)	
	Tuesday	70 (64–77)		58 (51–65)		12 (10–15)	
	Wednesday	73 (66–80)		58 (51–66)		15 (12–17)	
	Thursday	75 (67–84)		58 (51–67)		17 (14–21)	
	Friday	67 (61–74)		53 (47–60)		13 (11–16)	
	Saturday	73 (66–81)		59 (52–67)		14 (12–18)	
Total		71 (69–74)		56 (54–59)		15 (14–16)	

Appendix Table 4. Mean overall, household, and non-household close contact time (hours) in Western Cape. P-values were calculated using a Wald test.

Characteristic		Overall		Household members		Non-household members	
		Mean (95% CI)	p-value	Mean (95% CI)	p-value	Mean (95% CI)	p-value
Sex	Male	49 (46–53)	<0.001	27 (24–30)	<0.001	22 (20–25)	<0.3
	Female	60 (56–64)		40 (37–43)		20 (18–23)	
Age	15–19	80 (68–95)	<0.001	46 (39–53)	<0.001	35 (25–49)	0.006
	20–29	51 (47–56)		28 (25–32)		23 (19–27)	
	30–39	49 (45–53)		31 (28–34)		18 (16–21)	
	40–49	58 (51–66)		38 (32–46)		20 (16–25)	
	50+	63 (52–77)		44 (35–57)		19 (12–31)	
Residence	Peri-Urban	55 (52–57)		33 (31–35)		21 (19–24)	
	Urban	NA		NA		NA	
	Rural	NA		NA		NA	
Monthly household income	<R1000	58 (48–71)	0.001	37 (30–45)	<0.001	22 (16–30)	0.03
	R1000–R2500	54 (49–60)		35 (31–40)		19 (16–23)	
	R2500–R5000	60 (55–66)		34 (31–38)		26 (23–30)	
	R5000–R10000	41 (35–48)		24 (22–28)		16 (12–24)	
	>R10000	51 (42–62)		29 (25–35)		21 (14–33)	
	Unknown/missing	57 (51–64)		39 (33–46)		18 (13–24)	
Employment	Full-time	53 (49–58)	0.3	28 (25–31)	<0.001	26 (22–29)	0.005
	Part-time	52 (46–59)		31 (26–36)		21 (17–25)	
	None/missing	57 (53–62)		40 (36–43)		18 (15–21)	
Household size	1	27 (23–32)	<0.001	0.6 (0.3–1.3)	<0.001	26 (22–31)	0.009
	2–4	51 (48–54)		29 (28–31)		21 (19–24)	
	5–7	83 (78–88)		66 (62–70)		17 (13–22)	
	8–10	124 (109–141)		114 (97–134)	10 (5–21)		
	11+	200 (138–291)		172 (112–264)	28 (7–112)		
Day reported	Sunday	53 (46–60)	0.2	34 (30–52)	0.08	18 (14–23)	0.3
	Monday	60 (54–78)		34 (30–52)		26 (21–29)	
	Tuesday	52 (46–67)		32 (28–49)		20 (16–27)	
	Wednesday	57 (49–52)		32 (28–47)		24 (18–22)	
	Thursday	54 (48–66)		30 (26–43)		23 (19–28)	
	Friday	47 (40–45)		27 (23–33)		20 (14–19)	
	Saturday	60 (52–53)		42 (34–37)		18 (14–22)	
Total		55 (52–57)		33 (31–35)		21 (19–24)	

Appendix Table 5. Mean overall, household, and non-household casual contact time (hours) in KwaZulu-Natal. P-values were calculated using a Wald test.

Characteristic		Overall		Household members		Non-household members	
		Mean (95% CI)	p-value	Mean (95% CI)	p-value	Mean (95% CI)	p-value
Sex	Male	171 (158–185)	<0.001	86 (81–91)	<0.001	85 (73–99)	0.9
	Female	203 (191–216)		119 (114–125)		84 (73–97)	
Age	15–19	230 (195–270)	0.03	100 (86–115)	0.007	130 (98–172)	0.01
	20–29	204 (188–221)		108 (101–116)		95 (80–113)	
	30–39	182 (164–202)		105 (96–114)		77 (61–98)	
	40–49	170 (144–200)		91 (81–101)		79 (56–112)	
	50+	186 (170–204)		114 (107–122)		72 (57–91)	
Residence	Peri-Urban	177 (164–190)	0.03	98 (92–104)	<0.001	79 (67–92)	0.2
	Urban	203 (189–217)		117 (111–123)		86 (73–100)	
	Rural	186 (154–225)		74 (65–85)		112 (80–156)	
Monthly household income	<R1000	150 (136–166)	<0.001	91 (85–99)	<0.001	59 (46–74)	<0.001
	R1000–R2500	207 (193–222)		126 (119–132)		82 (69–97)	
	R2500–R5000	191 (170–214)		85 (77–94)		106 (85–131)	

Characteristic		Overall		Household members		Non-household members	
		Mean (95% CI)	p-value	Mean (95% CI)	p-value	Mean (95% CI)	p-value
	R5000–R10000	204 (170–245)		97 (83–113)		108 (76–152)	
	>R10000	222 (175–282)		91 (72–113)		132 (88–197)	
	Unknown/missing	204 (137–303)		69 (54–87)		135 (71–256)	
Employment	Full-time	188 (165–214)	0.9	87 (79–95)	<0.001	101 (80–129)	0.05
	Part-time	206 (148–286)		82 (66–101)		124 (72–213)	
	None/missing	191 (181–201)		112 (108–117)		78 (69–88)	
Household size	1	157 (124–198)	0.03	65 (56–77)	<0.001	91 (61–136)	0.4
	2–4	176 (152–204)		78 (71–87)		98 (76–127)	
	5–7	182 (164–201)		94 (89–100)		87 (71–108)	
	8–10	204 (187–224)		119 (111–128)		85 (68–106)	
	11+	207 (191–223)		136 (126–146)		71 (58–87)	
Day reported	Sunday	196 (170–225)	0.7	100 (91–110)	0.8	96 (72–127)	0.5
	Monday	177 (157–200)		106 (96–118)		70 (52–95)	
	Tuesday	191 (165–220)		113 (101–126)		78 (57–107)	
	Wednesday	194 (171–221)		106 (96–117)		88 (66–118)	
	Thursday	178 (159–200)		106 (95–118)		72 (55–95)	
	Friday	199 (176–226)		103 (94–113)		96 (74–125)	
	Saturday	199 (176–226)		108 (98–118)		91 (70–120)	
Total		191 (182–200)		106 (102–110)		85 (76–94)	

Appendix Table 6. Mean overall, household, and non-household casual contact time (hours) in Western Cape. P-values were calculated using a Wald test.

Characteristic		Overall		Household members		Non-household members	
		Mean (95% CI)	p-value	Mean (95% CI)	p-value	Mean (95% CI)	p-value
Sex	Male	142 (125–162)	0.2	38 (34–42)	<0.001	104 (88–124)	0.8
	Female	161 (146–179)		56 (52–62)		105 (90–122)	
Age	15–19	177 (154–204)	0.1	56 (50–64)	0.01	121 (98–148)	0.2
	20–29	141 (123–162)		44 (39–49)		98 (81–119)	
	30–39	162 (140–188)		44 (39–50)		119 (97–144)	
	40–49	141 (115–173)		51 (42–61)		90 (67–122)	
	50+	123 (83–182)		57 (44–74)		66 (34–131)	
Residence	Peri-Urban	151 (140–164)		47 (44–50)		105 (93–117)	
	Urban	NA		NA		NA	
	Rural	NA		NA		NA	
Monthly household income	<R1000	134 (105–171)	0.2	53 (39–73)	0.001	81 (55–118)	0.2
	R1000–R2500	160 (137–187)		56 (49–64)		105 (83–131)	
	R2500–R5000	166 (144–192)		44 (39–49)		122 (101–148)	
	R5000–R10000	131 (103–166)		36 (32–42)		95 (68–132)	
	>R10000	133 (103–173)		46 (37–58)		87 (59–128)	
	Unknown/missing	141 (115–172)		46 (39–55)		95 (70–129)	
Employment	Full-time	159 (138–182)	0.001	37 (33–42)	<0.001	122 (102–145)	<0.001
	Part-time	183 (152–221)		43 (35–52)		140 (111–178)	
	None/missing	128 (116–141)		59 (54–64)		69 (59–82)	
Household size	1	95 (73–123)	<0.001	12 (8–17)	<0.001	83 (62–112)	0.4
	2–4	154 (139–170)		42 (39–44)		113 (98–129)	
	5–7	186 (159–217)		88 (79–98)		98 (73–131)	
	8–10	235 (144–383)		133 (109–162)		102 (37–279)	
	11+	364 (183–722)		192 (114–325)		171 (35–829)	
Day reported	Sunday	150 (123–51)	0.9	49 (42–49)	0.9	100 (75–32)	0.9
	Monday	156 (130–55)		47 (41–59)		110 (85–36)	
	Tuesday	148 (122–50)		43 (37–55)		105 (80–34)	
	Wednesday	146 (120–50)		45 (39–52)		100 (75–31)	
	Thursday	137 (108–42)		46 (37–33)		90 (65–27)	
	Friday	149 (113–36)		48 (37–32)		101 (69–24)	
	Saturday	175 (141–47)		49 (39–34)		126 (93–32)	
Total		151 (140–164)		47 (44–50)		105 (93–117)	

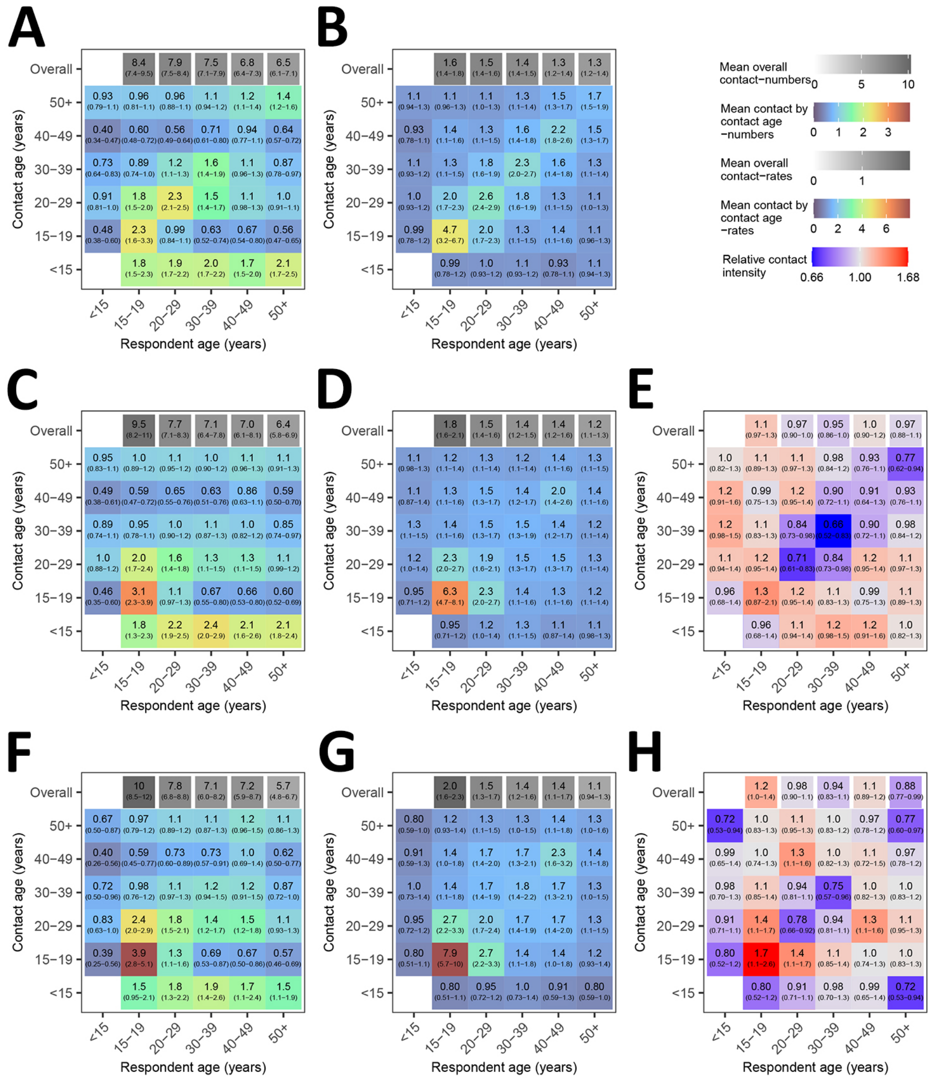

Appendix Figure 1. Age mixing matrices relevant for droplet transmission, non-saturating airborne transmission, and Mycobacterium tuberculosis transmission, for KwaZulu-Natal. Graphs a, c, and f show absolute contact intensities between respondents and contacts in each age group. Graphs b, d, and g show intensities of contact between each member of each age group. Graphs e and h show intensities for airborne infections and Mtb compared to intensities for droplet infections respectively. Numbers shown in graph a are the mean number of contacts respondents in each age group have with contacts in each age group per day. Numbers shown in graph b are the rate of contact between each individual in the population per day, expressed as rates ×105. 'Numbers' and 'rates' in graphs c, d, f, and g are standardized so that the mean overall contact intensity by reported by adult respondents is the same as the mean number of overall close contacts reported by adult respondents (graph a). Contact numbers between child 'respondents' and contacts in each age group were estimated from data on contact between adult respondents and child contacts. Ranges shown are bootstrapped 95% plausible ranges.

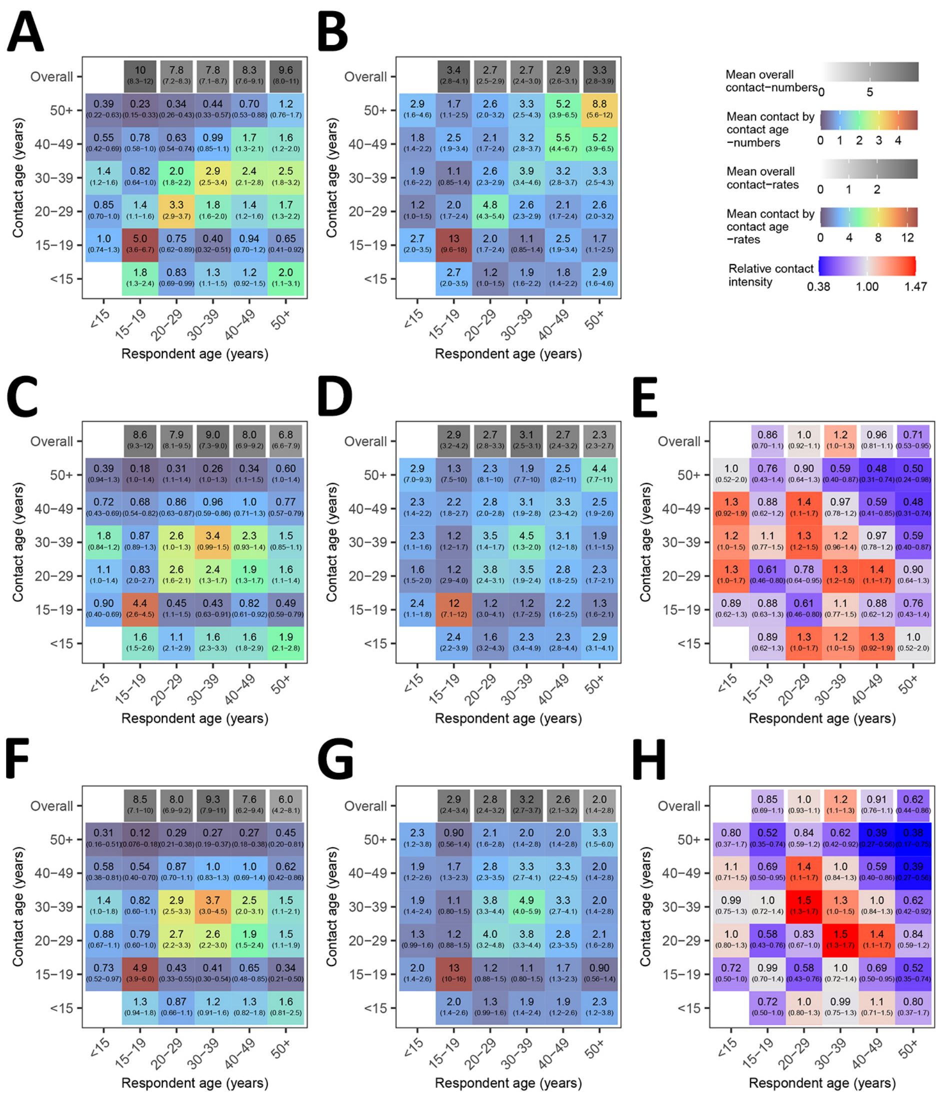

Appendix Figure 2. Age mixing matrices relevant for droplet transmission, non-saturating airborne transmission, and Mycobacterium tuberculosis transmission, for Western Cape. Graphs a, c, and f show absolute contact intensities between respondents and contacts in each age group. Graphs b, d, and g show intensities of contact between each member of each age group. Graphs e and h show intensities for airborne infections and Mtb compared to intensities for droplet infections respectively. Numbers shown in graph a are the mean number of contacts respondents in each age group have with contacts in each age group per day. Numbers shown in graph b are the rate of contact between each individual in the population per day, expressed as rates ×105. 'Numbers' and 'rates' in graphs c, d, f, and g are standardized so that the mean overall contact intensity by reported by adult respondents is the same as the mean number of overall close contacts reported by adult respondents (graph a). Contact numbers between child 'respondents' and contacts in each age group were estimated from data on contact between adult respondents and child contacts. Ranges shown are bootstrapped 95% plausible ranges.

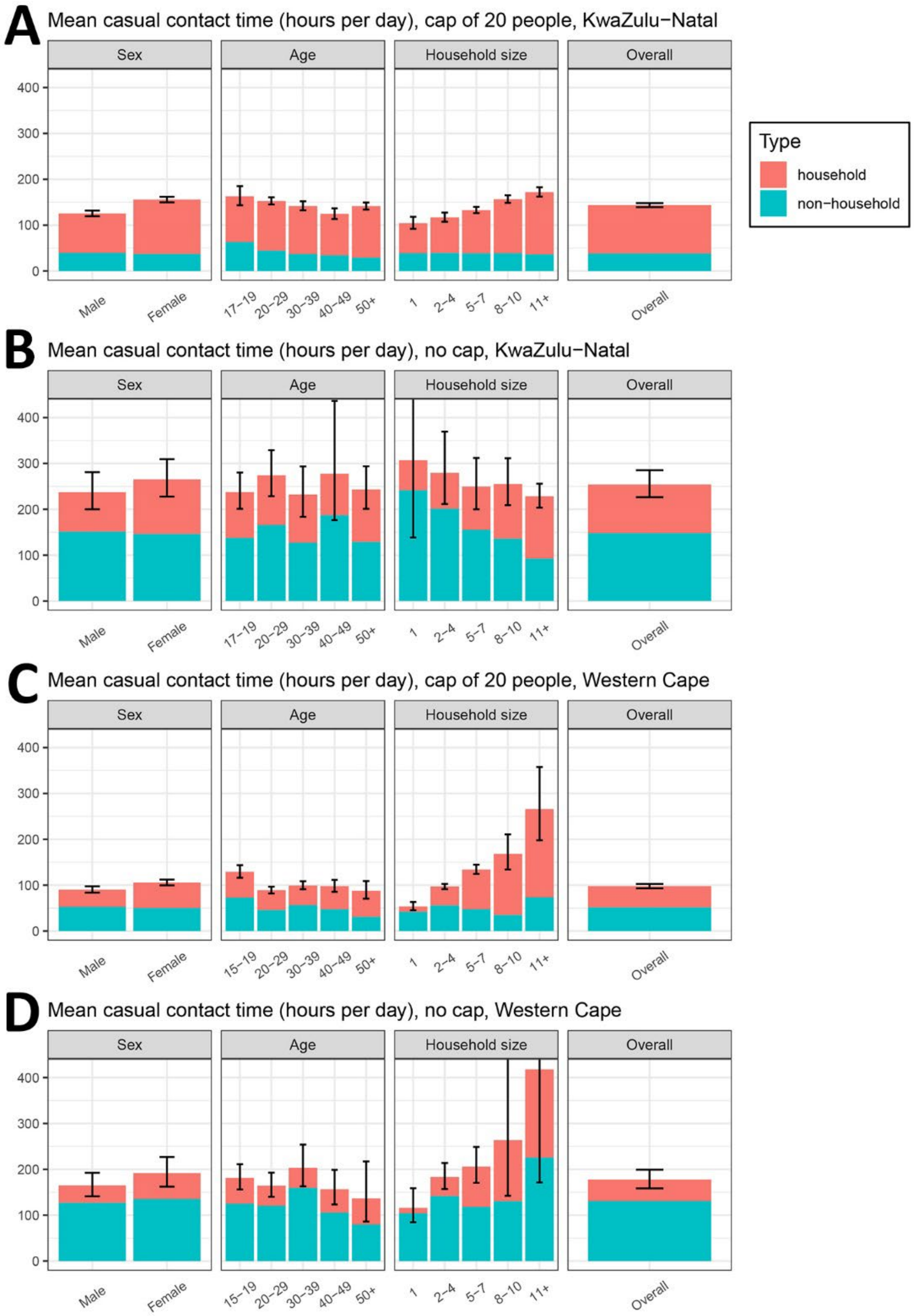

Appendix Figure 3. Household and non-household close contact numbers, close contact time, and casual contact time in KwaZulu-Natal and Western Cape, with a cap of 20 people at risk per location, and with no cap on the number at risk. Error bars show 95% confidence intervals for total contact numbers or time.

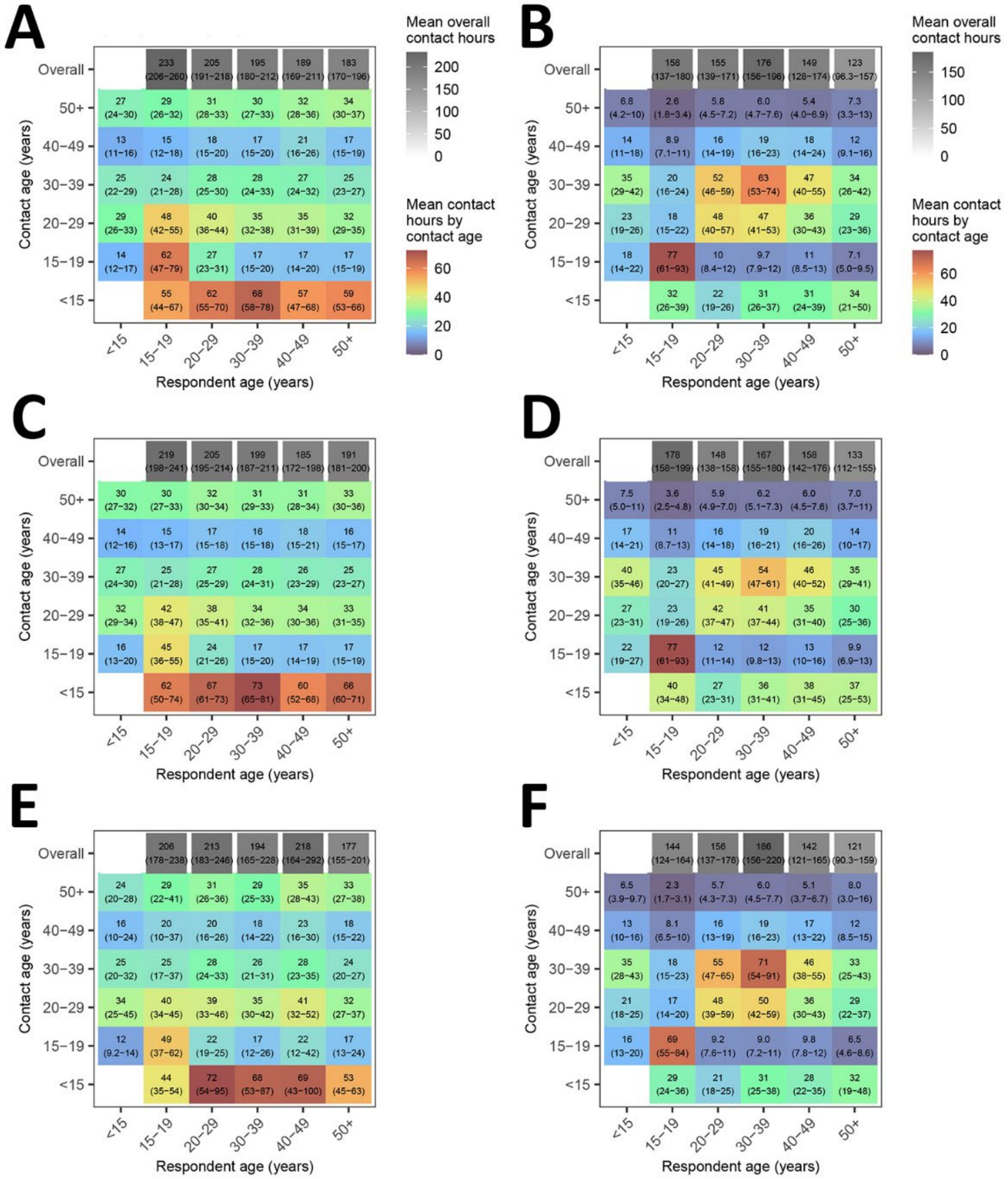

Appendix Figure 4. Casual contact time age mixing patterns for KwaZulu-Natal and Western Cape with a cap of 100 people at risk per location (baseline scenario), a cap of 20 people (sensitivity analysis), and no cap (sensitivity analysis). Panels a and b show estimated mean contact hours per day between respondents and contacts in each age group. Panels c-f show estimated mean contact hours, scaled to give the same overall mean adult contact hours per adult as panels a (c and e) and b (d and f).

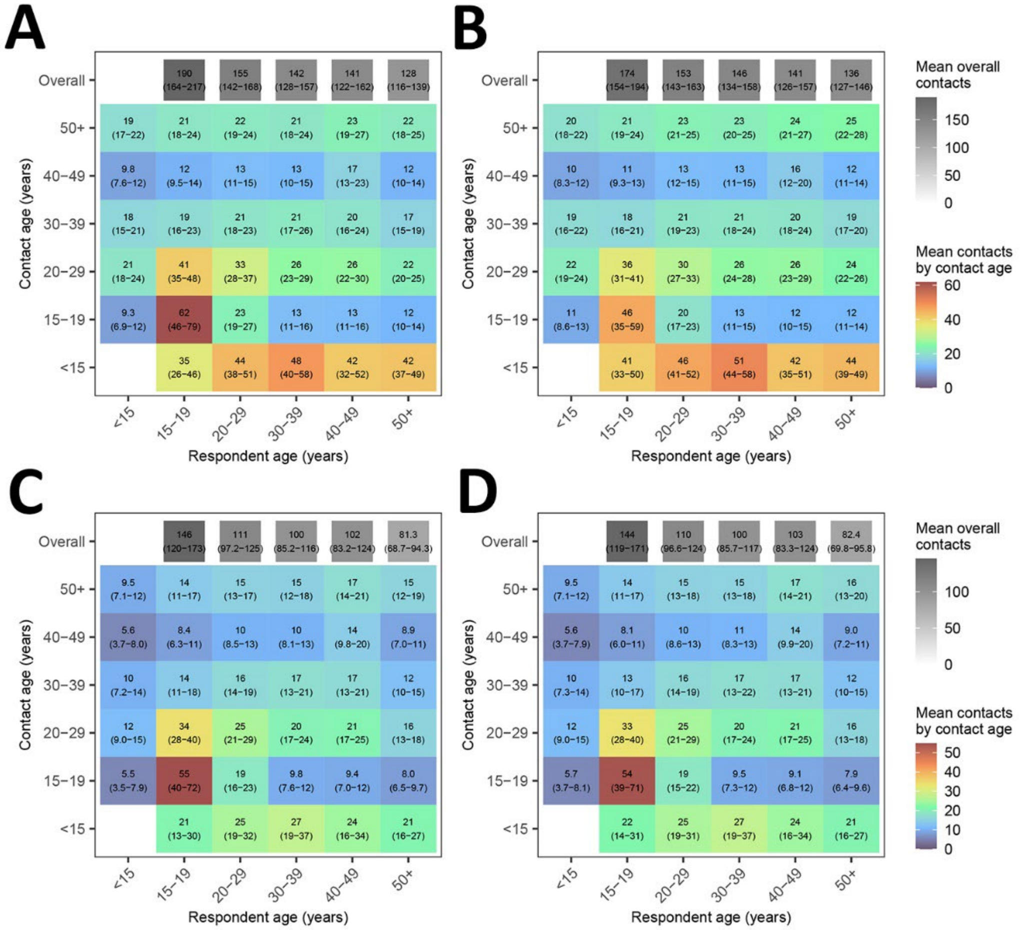

Appendix Figure 5. Age mixing patterns relevant for non-saturating airborne transmission and the transmission of *Mycobacterium* tuberculosis in KwaZulu-Natal, with household age mixing estimated using close (baseline) and casual (sensitivity analysis) contact data. Panels show mean contact hours per day between respondents and contacts in each age group. Numbers in b and d are scaled to give the same overall mean adult contact hours per adult as panels a and c respectively.

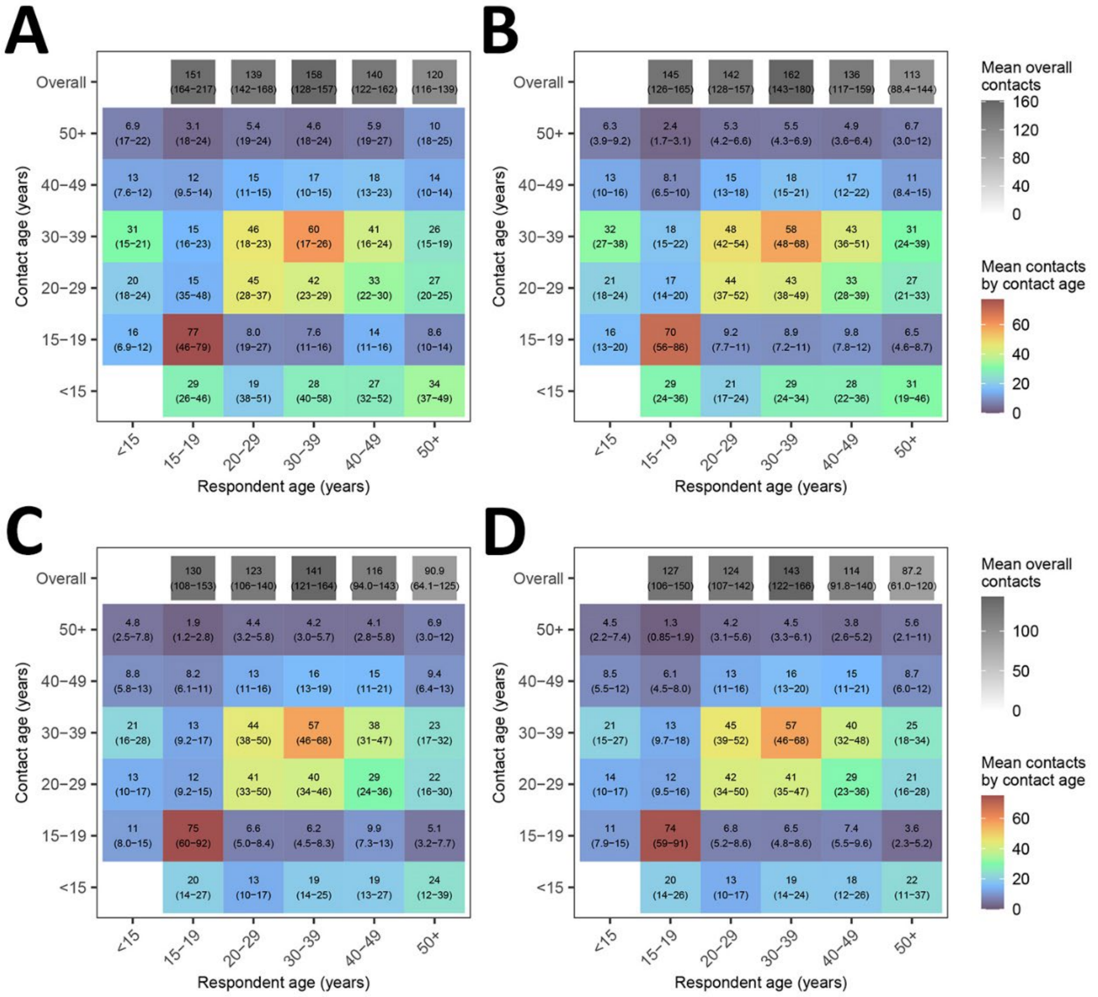

Appendix Figure 6. Age mixing patterns relevant for non-saturating airborne transmission and the transmission of *Mycobacterium* tuberculosis in Western Cape, with household age mixing estimated using close (baseline) and casual (sensitivity analysis) contact data. Panels show mean contact hours per day between respondents and contacts in each age group. Numbers in b and d are scaled to give the same overall mean adult contact hours per adult as panels a and c respectively of locations visited and transport used

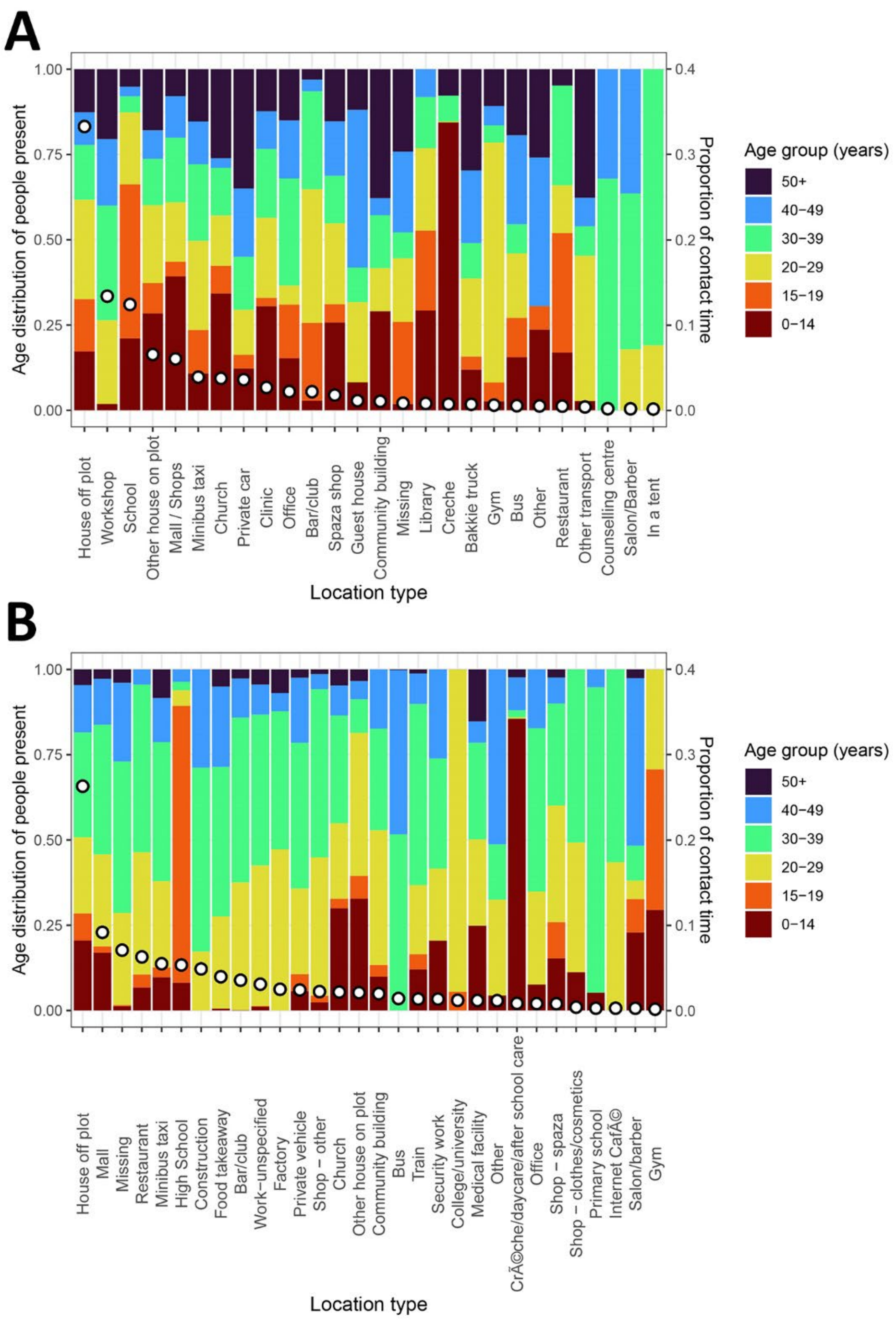

Appendix Figure 7. Age distribution of people present and proportion of contact time by location type, in KwaZulu-Natal and Western Cape. Bars (left axis) show the estimated proportion of people present in a location who are in each age group. White circles (right axis) show the estimated proportion of all contact time by adults in the community (excluding people's own homes) that occurs in each type of location.

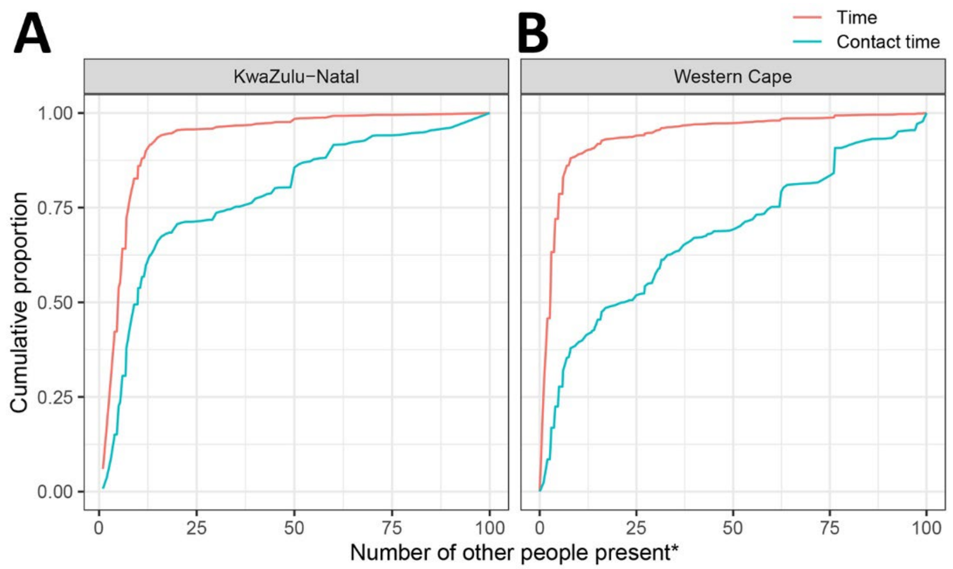

Appendix Figure 8. Cumulative proportion of time and casual contact time occurring in locations, by number of other people present, in KwaZulu-Natal and Western Cape. *Number of other people present is capped at 100

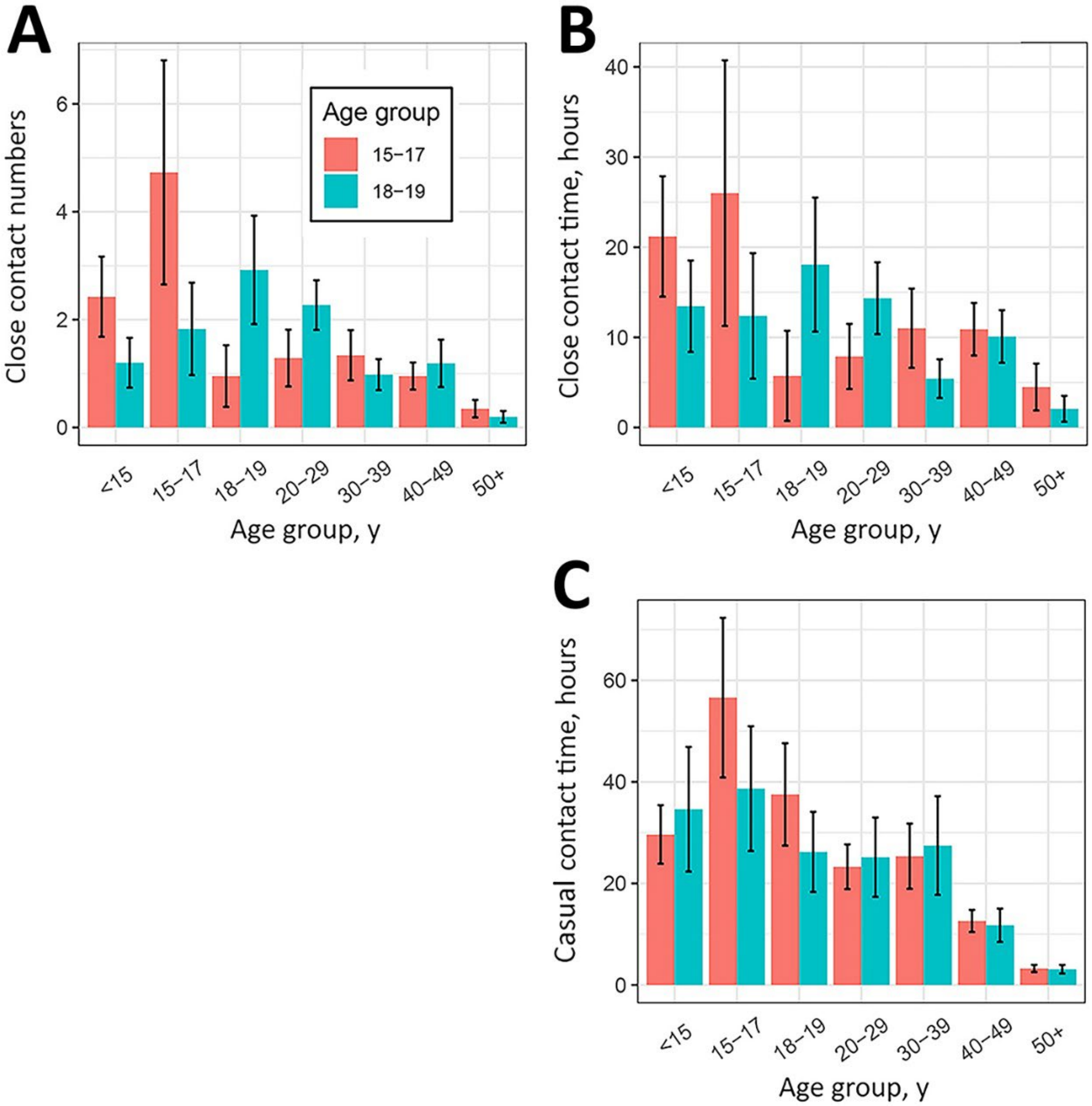

Appendix Figure 9. Adolescent contact patterns in Western Cape. Mean daily close contact numbers, close contact time, and casual contact time by contact age group for respondents aged 15–17 years and 18–19 years in Western Cape. Error bars show 95% confidence intervals.

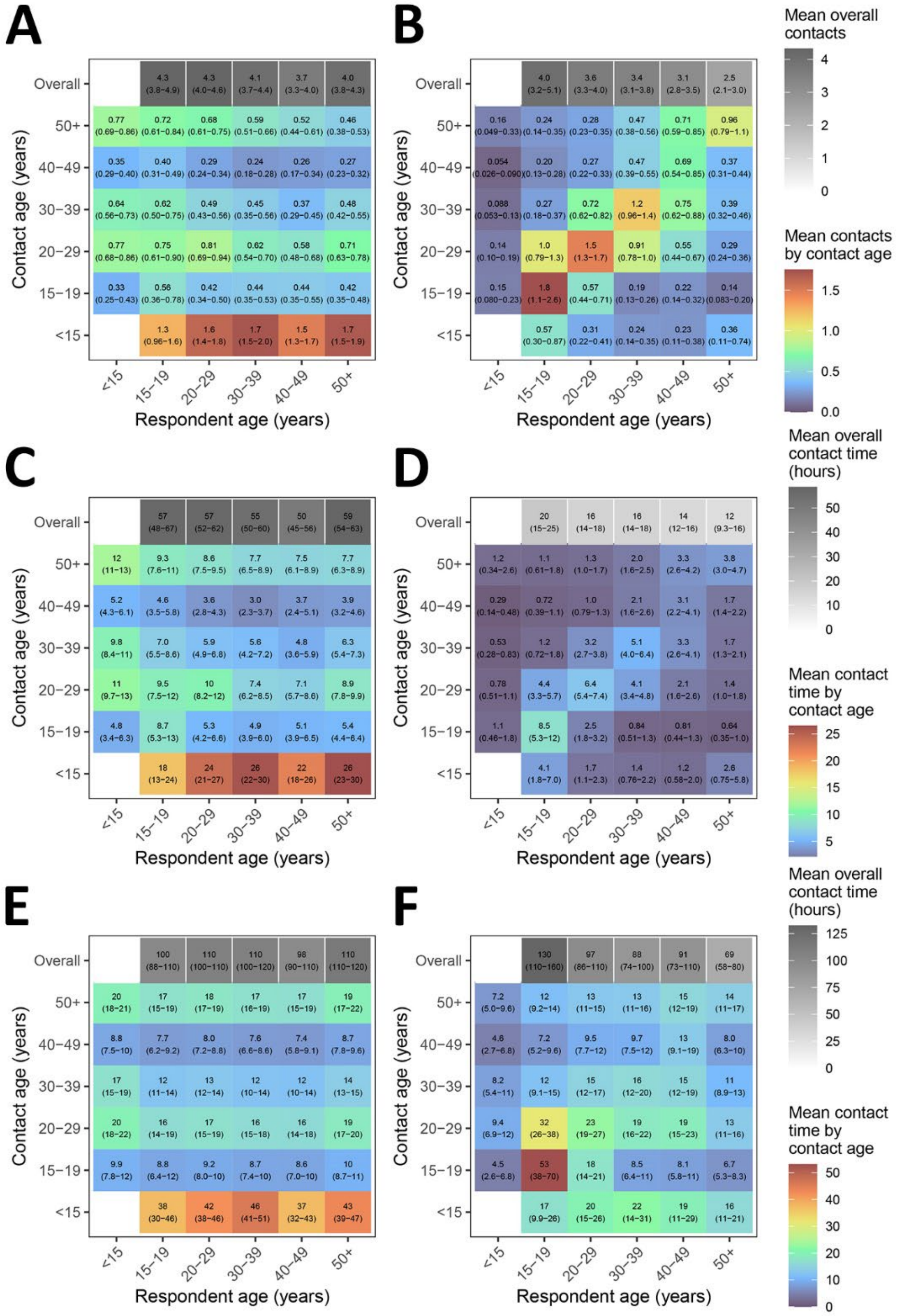

Appendix Figure 10. Household and non-household close contact numbers, close contact time, and casual contact time age-mixing matrices for KwaZulu-Natal. Numbers shown in graphs a and b are the mean number of contacts respondents in each age group have with contacts in each age group per day. Numbers shown in graphs c-f are the mean number of contact hours respondents in each age group have with contacts in each age group per day. Contact numbers between child 'respondents' and contacts in each age group were estimated from data on contact between adult respondents and child contacts. Ranges shown are bootstrapped 95% plausible ranges.

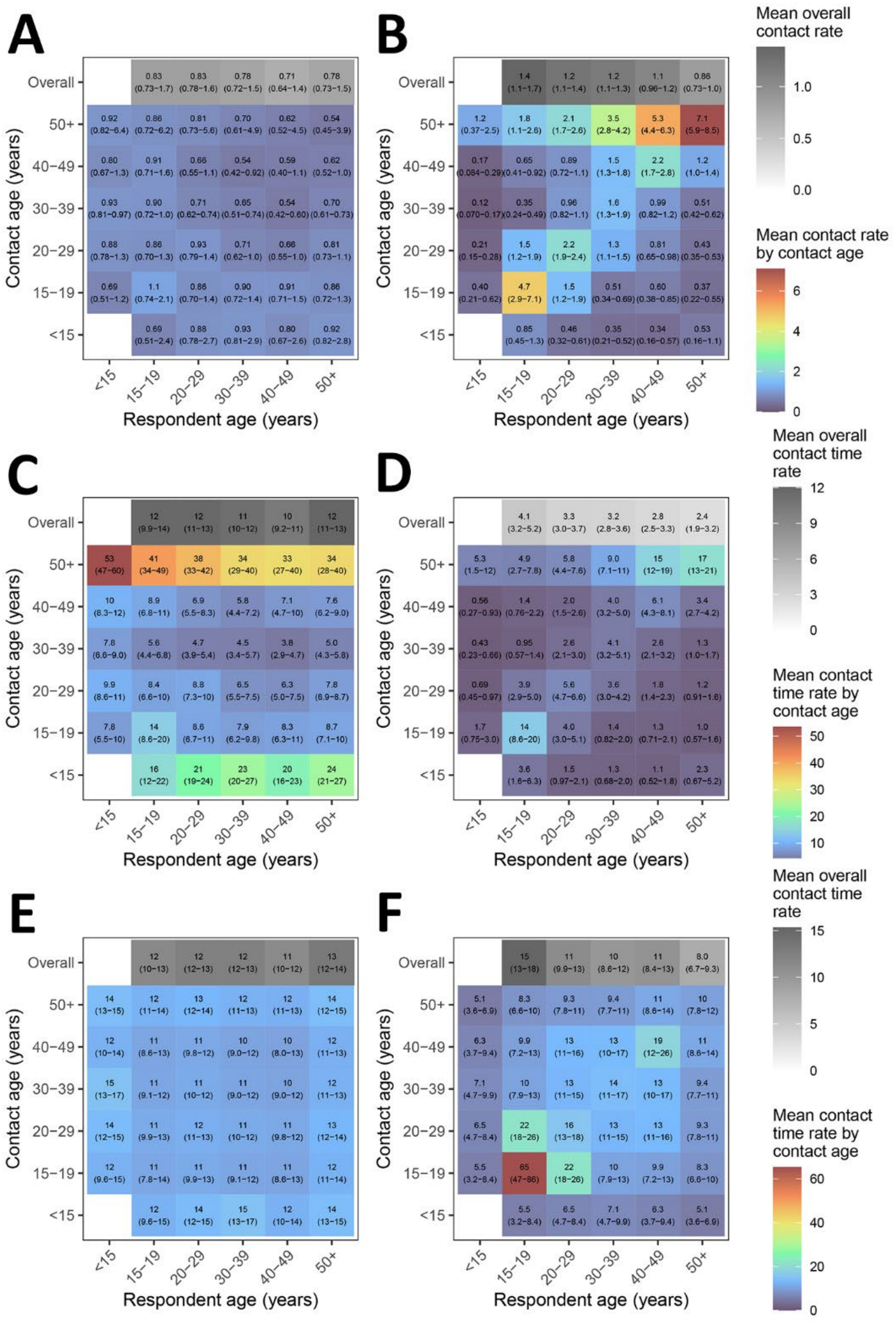

Appendix Figure 11. Household and non-household close contact numbers, close contact time, and casual contact time age-mixing matrices for KwaZulu-Natal. Numbers shown in graphs a and b are the rate of contact between each individual in the population per day, expressed as rates ×10^{5}. Numbers shown in graphs c-f are the mean minutes of contact between each individual in the population per day, expressed as rates ×10^{2}. Contact rates between child ‘respondents’ and contacts in each age group were estimated from data on contact between adult respondents and child contacts. Ranges shown are bootstrapped 95% plausible ranges.

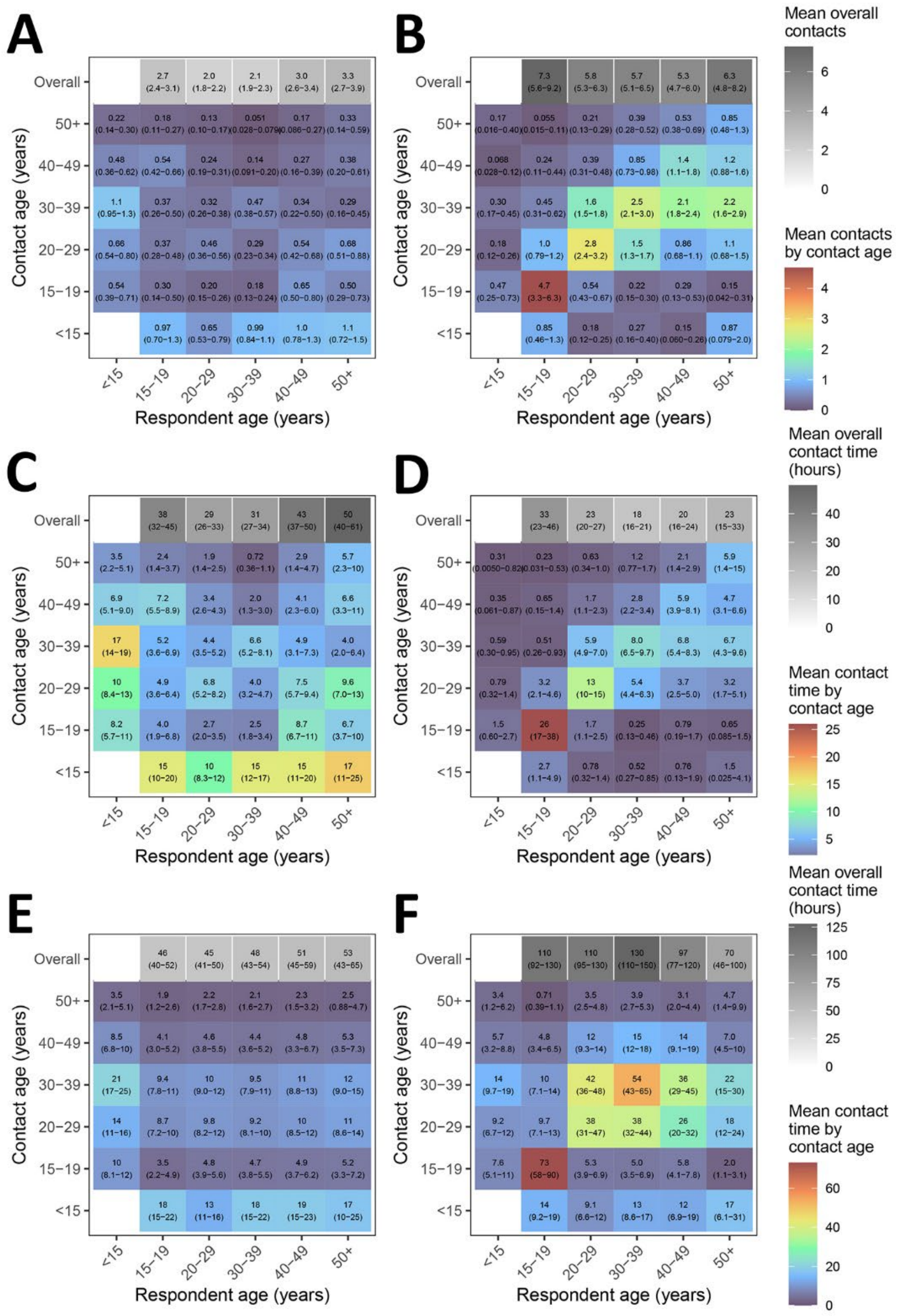

Appendix Figure 12. Household and non-household close contact numbers, close contact time, and casual contact time age-mixing matrices for Western Cape. Numbers shown in graphs a and b are the mean number of contacts respondents in each age group have with contacts in each age group per day. Numbers shown in graphs c-f are the mean number of contact hours respondents in each age group have with contacts in each age group per day. Contact numbers between child ‘respondents’ and contacts in each age group were estimated from data on contact between adult respondents and child contacts. Ranges shown are bootstrapped 95% plausible ranges.

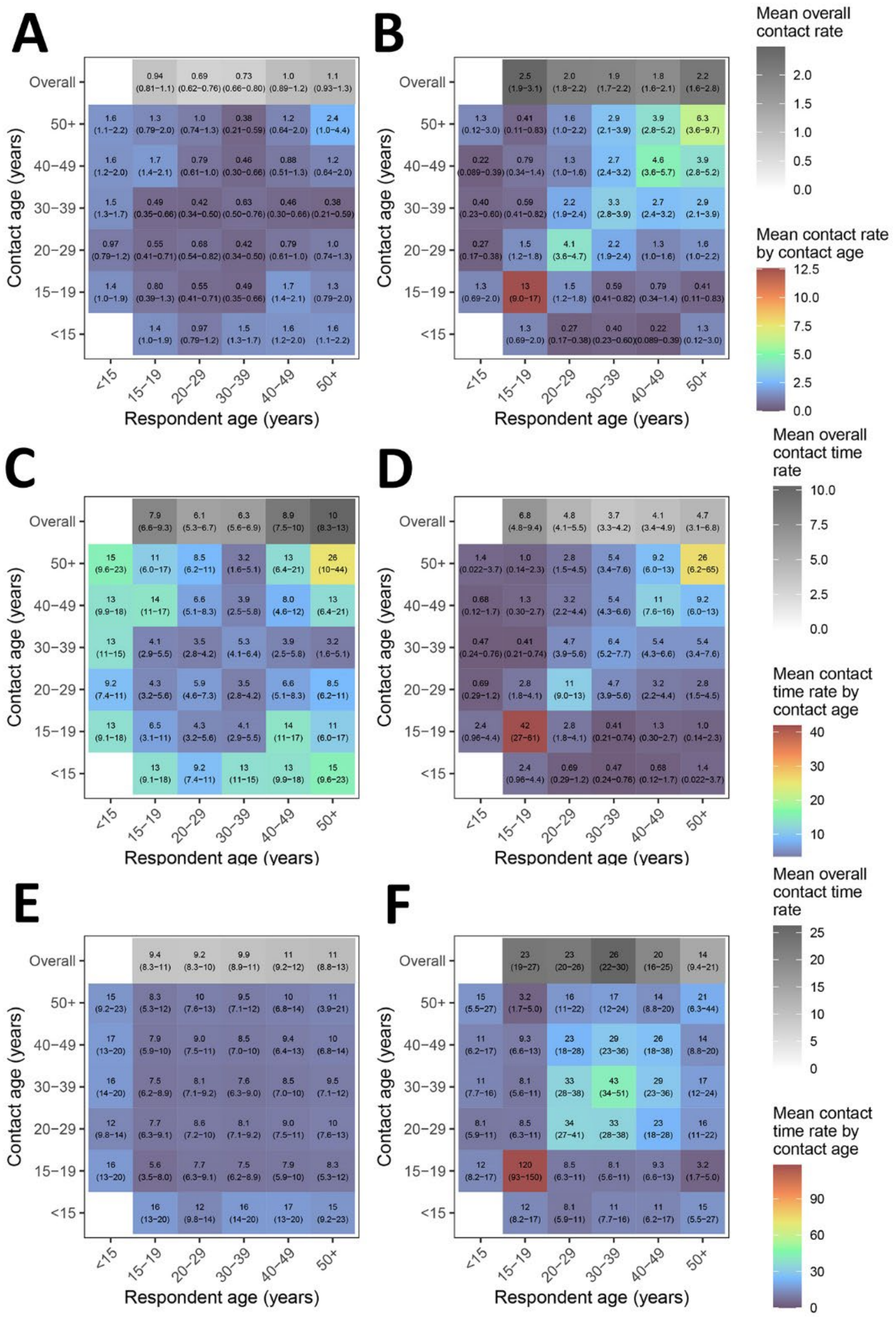

Appendix Figure 13. Household and non-household close contact numbers, close contact time, and casual contact time age-mixing matrices for Western Cape. Numbers shown in graphs a and b are the rate of contact between each individual in the population per day, expressed as rates $\times 10^5$. Numbers shown in graphs c-f are the mean minutes of contact between each individual in the population per day, expressed as rates $\times 10^2$. Contact rates between child ‘respondents’ and contacts in each age group were estimated from data on contact between adult respondents and child contacts. Ranges shown are bootstrapped 95% plausible ranges.
